# VEZT promotes pancreatic cancer progression by stabilizing SLC25A24 and preserving mitochondrial function

**DOI:** 10.3389/fimmu.2026.1900975

**Published:** 2026-08-19

**Authors:** Letian Chen, Zihan Yan, Zhihao Fang, Zhiping Yu, Ming Jiang, Yeqing Zou

**Affiliations:** 1 Jiangxi Key Laboratory of Molecular Medicine, The Second Affiliated Hospital, Jiangxi Medical College, Nanchang University, Nanchang, Jiangxi, China; 2 School of Artificial Intelligence and Information Engineering, East China University of Technology, Nanchang, Jiangxi, China; 3 Departments of Urology, Yingtan People’s Hospital, Yingtan, Jiangxi, China

**Keywords:** apoptosis, mitochondrial function, pancreatic cancer, SLC25A24, VEZT

## Abstract

**Background:**

Pancreatic cancer (PC) is a highly aggressive malignancy with an unclear pathogenesis, limited therapeutic options, and poor prognosis. Vezatin (VEZT), an adherens junction transmembrane protein, has been implicated in the development and progression of several malignancies, including gastric cancer. However, its biological role and underlying mechanisms in pancreatic cancer remain largely unexplored. This study aimed to investigate the role of VEZT in pancreatic cancer progression and elucidate the molecular mechanism by which VEZT regulates mitochondrial homeostasis through Solute Carrier Family 25 Member 24 (SLC25A24), thereby suppressing apoptosis and promoting malignant progression.

**Methods:**

The expression patterns and clinical significance of VEZT and SLC25A24 were analyzed using public datasets from TCGA and GTEx. Their biological functions were systematically evaluated through a series of *in vitro* experiments, including cell proliferation, migration, invasion, apoptosis, protein interaction, and mitochondrial function assays.

**Results:**

VEZT and SLC25A24 were both significantly upregulated in pancreatic cancer tissues, and their elevated expression was closely associated with unfavorable patient prognosis. Both molecules also exhibited good diagnostic performance. Immune infiltration analysis showed that the expression levels of VEZT and SLC25A24 were significantly associated with multiple tumor-infiltrating immune cell populations. Functional assays demonstrated that VEZT knockdown markedly inhibited pancreatic cancer cell proliferation, colony formation, migration, and invasion, while promoting apoptosis; restoration of SLC25A24 expression partially reversed these effects. Further investigations revealed a protein interaction between VEZT and SLC25A24. VEZT knockdown reduced the stability and expression level of SLC25A24 protein and was accompanied by impaired mitochondrial function.

**Conclusions:**

This study reveals the oncogenic role of VEZT in pancreatic cancer and identifies SLC25A24 as an important downstream effector. The VEZT/SLC25A24 axis may promote pancreatic cancer progression by modulating mitochondrial function and apoptosis, highlighting its potential value for prognostic assessment and therapeutic targeting.

## 1 Introduction

Pancreatic cancer (PC) is one of the most aggressive malignancies of the digestive system and remains a major cause of cancer-related mortality worldwide. Owing to its insidious onset and lack of specific early clinical symptoms, most patients are diagnosed at an advanced stage, resulting in limited therapeutic opportunities and poor clinical outcomes. Despite advances in surgical techniques, chemotherapy, targeted therapy, and immunotherapy, the prognosis of pancreatic cancer remains dismal, with a median survival time of approximately 4 months and a 5-year overall survival rate of less than 15% (1). Therefore, elucidating the molecular mechanisms underlying pancreatic cancer progression and identifying novel therapeutic targets are of critical importance for improving patient outcomes.

Vezatin (VEZT) is a transmembrane protein originally identified at adherens junctions, where it interacts with the cadherin–catenin complex and the actin cytoskeleton to maintain cell–cell adhesion, cytoskeletal organization, and tissue integrity (2, 3). Notably, the subcellular distribution of VEZT is not restricted to the plasma membrane. Previous studies have shown that different VEZT isoforms may be enriched at intercellular junctions, in the cytoplasm, nucleus, and cytoplasmic vesicular structures, suggesting that VEZT may exert intracellular functions beyond its canonical role in adherens junctions (4). Immunofluorescence data from the Human Protein Atlas also indicate that VEZT can localize to the nucleoplasm and cytosol, further supporting the existence of an intracellular VEZT pool with potential noncanonical functions.

Accumulating evidence indicates that aberrant VEZT expression is closely associated with the initiation and progression of multiple malignancies. Previous studies have implicated VEZT in the malignant progression of gastric cancer and suggested that it may be involved in the regulation of tumor cell proliferation, migration, and invasion (5, 6) However, the expression profile, clinical significance, and biological functions of VEZT in pancreatic cancer have not been systematically characterized. Moreover, whether VEZT regulates pancreatic cancer cell survival and malignant progression through specific intracellular effector molecules remains unclear.

Mitochondrial dysfunction is a key feature of tumor initiation and progression. In addition to their central role in energy production and metabolic adaptation, mitochondria are critically involved in redox regulation, ion homeostasis, and intrinsic apoptotic signaling (7). Solute carrier family 25 member 24 (SLC25A24) is a member of the mitochondrial inner membrane carrier family that participates in ATP/Ca^2+^ transport and mitochondrial ion homeostasis and is closely associated with mitochondrial membrane potential, energy metabolism, and cellular stress responses (8). Although SLC25A24 may contribute to metabolic adaptation and survival in tumor cells, its biological significance in pancreatic cancer and its potential regulation by VEZT remain poorly understood.

Against this background, the present study systematically investigated the expression patterns, clinical significance, and prognostic value of VEZT and SLC25A24 in pancreatic cancer. Single-cell RNA sequencing was further employed to characterize their cellular distribution within the pancreatic cancer microenvironment. *In vitro* functional assays were subsequently performed to evaluate the effects of VEZT and SLC25A24 on pancreatic cancer cell proliferation, migration, invasion, and apoptosis, followed by analyses of their protein association and regulatory relationship. Our findings showed that both VEZT and SLC25A24 were upregulated in pancreatic cancer and suggested that VEZT may promote malignant progression by maintaining SLC25A24 protein stability, thereby modulating mitochondrial function and apoptosis. These findings provide new insights into the noncanonical intracellular role of VEZT in pancreatic cancer and suggest that the VEZT/SLC25A24 axis may serve as a potential target for prognostic assessment and therapeutic intervention.

## 2 Materials and methods

### 2.1 Bioinformatics analysis

#### 2.1.1 Expression profile of VEZT across human cancers and normal tissues

The expression pattern of VEZT across 33 human cancer types and their corresponding normal tissues was analyzed using the GEPIA2 platform, which integrates transcriptomic data from The Cancer Genome Atlas (TCGA) and the Genotype-Tissue Expression (GTEx) databases. In addition, protein expression characteristics of VEZT in different normal human tissues and cell types were investigated using data obtained from the Human Protein Atlas (HPA) database (https://www.proteinatlas.org/).

#### 2.1.2 Pan-cancer immune correlation and overall survival analysis of VEZT

The correlations between VEZT expression and immune cell infiltration across multiple cancer types were analyzed using the ggplot2 package (version 3.4.4) in R. To evaluate the prognostic significance of VEZT in pan-cancer, transcriptomic expression profiles and corresponding clinical information were obtained from the TCGA database. Samples lacking survival time or survival status information were excluded. Kaplan–Meier survival analysis and univariate Cox proportional hazards regression analysis were performed using the “survival” and “survminer” packages in R (version 4.3.0) to investigate the association between VEZT expression and overall survival (OS).

#### 2.1.3 Expression and prognostic analysis of VEZT and SLC25A24 in pancreatic cancer

The mRNA and protein expression levels of VEZT and SLC25A24 in pancreatic cancer tissues were analyzed based on data from the TCGA and GTEx databases. Patients were divided into high- and low-expression groups according to the optimal cutoff value. Survival differences between groups were assessed using the log-rank test and Cox regression analysis. Hazard ratios (HRs), 95% confidence intervals (CIs), and corresponding P values were calculated. A P value < 0.05 was considered statistically significant.

#### 2.1.4 Diagnostic value, survival prognosis, and clinical correlation analysis of VEZT and SLC25A24

Receiver operating characteristic (ROC) curve analysis was performed to evaluate the diagnostic performance of VEZT and SLC25A24 in pancreatic cancer. Sample status was used as the classification variable, whereas gene expression levels were used as predictive variables. Diagnostic accuracy was assessed by calculating the area under the curve (AUC).

To further investigate the prognostic significance of VEZT and SLC25A24, pancreatic cancer transcriptomic and clinical data were obtained from the TCGA database. Samples with incomplete survival information were excluded. Kaplan–Meier survival analysis and univariate Cox regression analysis were conducted to evaluate the associations of VEZT and SLC25A24 expression with overall survival (OS) and progression-free survival (PFS). Univariate and multivariate Cox proportional hazards models were subsequently constructed to assess their independent prognostic value after adjustment for clinical characteristics. The results were visualized using forest plots.

Furthermore, the associations between VEZT expression and clinicopathological features, including T stage, histological grade, pathological stage, and tumor subtype, were analyzed using the “limma” and “ggpubr” packages in R. Differences between groups were evaluated using the Wilcoxon rank-sum test and visualized using boxplots.

#### 2.1.5 Correlation analysis and molecular docking between VEZT and SLC25A24

Pearson correlation analysis was performed to evaluate the potential regulatory relationship between VEZT and SLC25A24 in pancreatic cancer. To further investigate their potential protein–protein interaction, molecular docking analysis was conducted using the HDOCK online platform. Potential binding conformations were searched using a fast Fourier transform (FFT)-based docking algorithm and evaluated using a knowledge-based scoring function. The docking conformation with the lowest binding energy score was selected as the optimal binding model and visualized using PyMOL software (9).

#### 2.1.6 Correlation of SLC25A24 with the tumor microenvironment and immune checkpoint genes

Samples were divided into high- and low-expression groups according to the median expression level of SLC25A24. Differences in tumor microenvironment (TME) scores between groups were compared using the Wilcoxon rank-sum test. Correlation analyses were subsequently performed to assess the relationships between VEZT expression and immune cell infiltration levels, and the results were visualized using lollipop plots.

Pearson correlation analysis was further conducted to evaluate the associations of VEZT and SLC25A24 with immune checkpoint genes. Correlations with P values < 0.001 were considered significant. Correlation matrices were constructed and visualized using the “corrplot” package in R (10).

#### 2.1.7 Diagnostic and prognostic analyses of the VEZT/SLC25A24 combined model

A combined diagnostic model based on VEZT and SLC25A24 expression was established using logistic regression analysis according to the following formula:


Risk Score = −9.902200 + 1.067189 × VEZT + 1.899521 × SLC25A24。

The diagnostic performance of the combined model was compared with those of single-gene models and ratio-based models (11) Model robustness was evaluated using 100 iterations of five-fold cross-validation. ROC curves and AUC values were calculated to assess diagnostic performance, and the optimal cutoff value was determined using the Youden index (12, 13).

Decision curve analysis (DCA) was performed to evaluate the clinical utility of the model. Patients were stratified into different risk groups according to risk score tertiles. Kaplan–Meier survival curves were generated, and survival differences among groups were compared using the log-rank test (14). Statistical analyses were performed using Python 3.9 with the scikit-learn, scipy, and lifelines packages. A P value < 0.05 was considered statistically significant.

#### 2.1.8 Single-cell analyses of VEZT and SLC25A24

Single-cell RNA sequencing (scRNA-seq) data (GSE205013) and spatial transcriptomic data (GSE235315) were downloaded from the Gene Expression Omnibus (GEO) database.

For scRNA-seq analysis, quality-control filtering criteria were set as follows: mitochondrial gene percentage <20%, number of detected genes between 200 and 6,000, and UMI counts >500. Data normalization was performed using the LogNormalize method implemented in the Seurat package (version 4.0), followed by principal component analysis (PCA). Uniform Manifold Approximation and Projection (UMAP) was performed using the top 30 principal components for dimensionality reduction and clustering. Twelve major cell populations were identified based on canonical marker genes. Cell–cell communication analysis was conducted using the CellPhoneDB algorithm to identify significant ligand–receptor interactions between different cell types (15, 16).

### 2.2 Patients and tissue specimens

A total of 31 pancreatic cancer tissue specimens and matched adjacent non-tumorous tissues were collected from patients who underwent radical pancreatic cancer resection at the Department of General Surgery, the Second Affiliated Hospital of Nanchang University. None of the patients had received radiotherapy, chemotherapy, targeted therapy, or immunotherapy prior to surgery. Tumor tissues and corresponding adjacent normal tissues were obtained intraoperatively and subsequently confirmed by pathological examination for further analyses. This study was approved by the Ethics Committee of the Second Affiliated Hospital of Nanchang University. The requirement for written informed consent from all participants was waived by the Ethics Committee. All procedures were conducted in accordance with the ethical principles of the Declaration of Helsinki.

### 2.3 Cell culture and lentiviral transduction

The human normal pancreatic ductal epithelial cell line HPDE6-C7 and human pancreatic cancer cell lines PANC-1, SW1990, AsPC-1, and BxPC-3 were purchased from Wuhan Procell Life Science & Technology Co., Ltd. (Wuhan, China). All cell lines were authenticated by short tandem repeat (STR) profiling and confirmed to be free of mycoplasma contamination.

Cells were cultured in complete medium supplemented with 10% fetal bovine serum (FBS)(Thermo Fisher Scientific, Waltham, MA, USA) and maintained at 37 °C in a humidified incubator containing 5% CO_2_.shRNA constructs targeting VEZT and SLC25A24, as well as corresponding overexpression vectors, were generated by Sangon Biotech (Shanghai) Co., Ltd. (Shanghai, China). Cell transfection was performed using PEI Transfection Reagent (Cat. No. G1802, Servicebio, Wuhan, China)according to the manufacturer’s instructions.

For lentiviral packaging, 293T cells( Procell Life Science & Technology Co., Ltd., Wuhan, China)were seeded into six-well plates and transfected when cell confluence reached 80–90%. PMDL (1.16 μg), PVSVG (0.62 μg), PREV (0.45 μg), and the corresponding plasmids were co-transfected into each well in a total volume of 2 mL. After 48 h, viral supernatants were collected, centrifuged at 300–500 × g for 5 min to remove cell debris, and filtered through a 0.45-μm membrane to obtain lentiviral particles (17). Target cells were infected with lentivirus for 48–72 h, and transduction efficiency was verified by Western blot analysis of VEZT(Cat. No. Ag13114, Proteintech Group, Inc., Wuhan, China) and SLC25A24(Cat. No.14669-1-AP, Proteintech Group, Inc., Wuhan, China) expression levels.

### 2.4 Quantitative real-time PCR

Cells were harvested when confluence reached 70–90%, and total RNA was extracted using an RNA Rapid Purification Kit (Beyotime Biotechnology, Shanghai, China). Complementary DNA (cDNA) was synthesized using the PrimeScript™ RT Reagent Kit (Takara Bio Inc., Shiga, Japan). Quantitative real-time PCR was performed using TB Green^®^ Premix Ex Taq™ II(Takara Bio Inc., Shiga, Japan), with GAPDH serving as the internal reference gene. Relative gene expression levels were calculated using the 2^-ΔΔCt method. All primers were synthesized by Sangon Biotech (Shanghai) Co., Ltd. (Shanghai, China). The primer sequences were as follows: VEZT forward, 5′-ACCGAAGTGATTTCCAGAGG-3′, and reverse, 5′-AGATGCTGACTTGGATGCTG-3′; SLC25A24 forward, 5′-CCCCTTTGGACCGTCTGA-3′, and reverse, 5′-AGCGAGCGGATACCTCCTT-3′; and GAPDH forward, 5′-GGAGCGAGATCCCTCCAAAAT-3′, and reverse, 5′-GGCTGTTGTCATACTTCTCATGG-3′.

### 2.5 Western blot analysis

Cells were washed twice with phosphate-buffered saline (PBS) and collected by centrifugation at 10,000 × g for 10 min at 4 °C. Total protein was extracted using RIPA lysis buffer (Solarbio Science & Technology Co., Ltd., Beijing, China), and protein concentrations were determined using a BCA Protein Assay Kit (Solarbio Science & Technology Co., Ltd., Beijing, China).

Equal amounts of protein (20 μg) were mixed with loading buffer, denatured at 100 °C for 10 min, separated by 10% SDS-PAGE(Epizyme Biomedical Technology Co., Ltd., Shanghai, China), and transferred onto nitrocellulose (NC) membranes. Membranes were blocked with 5% non-fat milk for 2 h at room temperature and incubated overnight at 4 °C with primary antibodies against VEZT, SLC25A24, and GAPDH(Cat. No.10494-1-AP, Proteintech Group, Inc., Wuhan, China). Following three washes with TBST(Servicebio, Wuhan, China), membranes were incubated with HRP-conjugated secondary antibodies (Cat. No.SA00001-2, Proteintech Group, Inc., Wuhan, China)for 1 h at room temperature.

Protein bands were visualized using enhanced chemiluminescence (ECL) (UElandy, Suzhou, China)reagents and quantified using Image Lab software (Bio-Rad, USA). All experiments were independently repeated at least three times.

### 2.6 Co-immunoprecipitation

Co-immunoprecipitation assays were performed to verify the interaction between VEZTand SLC25A24. Cells were lysed in IP lysis buffer supplemented with protease inhibitors, and lysates were centrifuged to collect the supernatant. Equal amounts of protein lysates were incubated overnight at 4 °C with anti-VEZT or anti-SLC25A24 antibodies, followed by incubation with Protein A/G magnetic beads (Beyotime Biotechnology, Shanghai, China). After three washes, the immunoprecipitated complexes were collected and analyzed by Western blotting (18).

### 2.7 Confocal immunofluorescence and protein colocalization analysis

Treated PANC-1 and SW1990 cells were seeded in confocal imaging dishes. After cell attachment, the cells were fixed with 4% paraformaldehyde and permeabilized with 0.3% Triton X-100. The cells were then blocked with 5% bovine serum albumin and incubated overnight at 4 °C with primary antibodies against VEZT (Cat. No. YP-mAb-09927; UpingBio Biotechnology Co., Ltd., Shenzhen, China) and SLC25A24. On the following day, the cells were incubated with the appropriate fluorescence-conjugated secondary antibodies in the dark, followed by nuclear counterstaining with DAPI. Images were acquired using a confocal laser-scanning microscope to assess the intracellular localization and colocalization of VEZT and SLC25A24.

### 2.8 Cell proliferation assays

#### 2.8.1 Colony formation assay

Cells were digested into single-cell suspensions and seeded into six-well plates at a density of 500–1000 cells per well. Three replicate wells were included for each group. The culture medium was replaced every 3–4 days. At the end of the incubation period, colonies were fixed with 4% paraformaldehyde (Solarbio Science & Technology Co., Ltd., Beijing, China)for 20 min and stained with 0.1% crystal violet(Solarbio Science & Technology Co., Ltd., Beijing, China) for 15–30 min. Colonies were photographed and quantified using ImageJ software.

#### 2.8.2 EdU incorporation assay

Cell proliferative activity was assessed using the YF^®^488 EdU Cell Proliferation Kit (UElandy, Suzhou, China). The assay was performed according to the manufacturer’s instructions. After incubation with Apollo reaction solution, nuclei were counterstained with Hoechst 33342. Fluorescence images were acquired using a fluorescence microscope, and the proportion of EdU-positive cells was quantified using ImageJ software.

### 2.9 Cell migration assays

#### 2.9.1 Wound-healing assay

Cells were seeded into six-well plates at a density of 4 × 10^5^ cells per well. When cell confluence reached approximately 90%, a linear scratch was generated using a sterile 200-μL pipette tip. Detached cells were removed by washing with PBS, and the cells were cultured in serum-free medium. Images were captured at 0 h and 24 h. Migration rates were calculated by measuring wound closure using ImageJ software.

#### 2.9.2 Transwell migration assay

A total of 2 × 10^4^ cells suspended in serum-free medium were seeded into the upper chamber of Transwell inserts (8-μm pore size), whereas medium (Solarbio Science & Technology Co., Ltd., Beijing, China)supplemented with 10% FBS was added to the lower chamber as a chemoattractant. After 24 h of incubation, non-migrated cells on the upper membrane surface were removed using cotton swabs. Migrated cells were fixed with 4% paraformaldehyde, stained with crystal violet, photographed under a microscope, and quantified using ImageJ software.

### 2.10 Protein stability assay

Cycloheximide (CHX; HY-12320, MedChemExpress, USA) chase assays were performed to evaluate the effect of VEZT on SLC25A24 protein stability. When cell confluence reached approximately 80%, cells were treated with CHX at a final concentration of 100 μg/mL and harvested at 0, 4, 8, and 12 h after treatment (19). Total protein was extracted, and SLC25A24 protein expression at different time points was analyzed by Western blotting to determine its degradation rate.

### 2.11 Apoptosis analysis

Cell apoptosis was assessed using an Annexin V-FITC/PI Apoptosis Detection Kit (UElandy, Suzhou, China). Cells were collected and stained according to the manufacturer’s instructions, followed by incubation at room temperature in the dark for 15 min. Samples were analyzed within 1 h using a Beckman Coulter CytoFLEX S flow cytometer. Data were processed using CytExpert 2.4 software, and the percentages of early and late apoptotic cells were calculated (20).

### 2.12 Mitochondrial fluorescence staining

MitoTracker Red CMXRos(Beyotime Biotechnology, Shanghai, China), MitoTracker Green FM(Beyotime Biotechnology, Shanghai, China), and MitoSOX Red (Beyotime Biotechnology, Shanghai, China) were used to assess mitochondrial membrane potential, mitochondrial mass, and mitochondrial reactive oxygen species (mtROS), respectively. Cells were stained according to the manufacturer’s instructions and subsequently imaged using a confocal laser scanning microscope. The fluorescence intensity of MitoTracker Red CMXRos is dependent on mitochondrial membrane potential and was used as an indicator of mitochondrial functional status. MitoTracker Green FM fluorescence was used to estimate mitochondrial mass, whereas MitoSOX Red fluorescence was used to evaluate mitochondrial superoxide production. Additionally, the expression level of the mitochondrial outer membrane protein TOM20 (Cat. No. 66777-1-Ig, Proteintech Group, Inc., Wuhan, China)was assessed by Western blotting to further evaluate mitochondrial content.

### 2.13 Statistical analysis

All experiments were independently repeated at least three times. Data are presented as the mean ± standard error of the mean (SEM). Statistical analyses were performed using GraphPad Prism 8.0 and R software.

Comparisons between two groups were conducted using Student’s t-test, whereas comparisons among multiple groups were performed using one-way analysis of variance (ANOVA) followed by Tukey’s multiple-comparison test. Correlations between variables were analyzed using Pearson’s correlation analysis or Spearman’s rank correlation analysis as appropriate. All statistical tests were two-sided, and a P value < 0.05 was considered statistically significant.

## 3 Results

### 3.1 Expression pattern of VEZT across human cancers and its association with immune infiltration and prognosis

**Figure 1:**
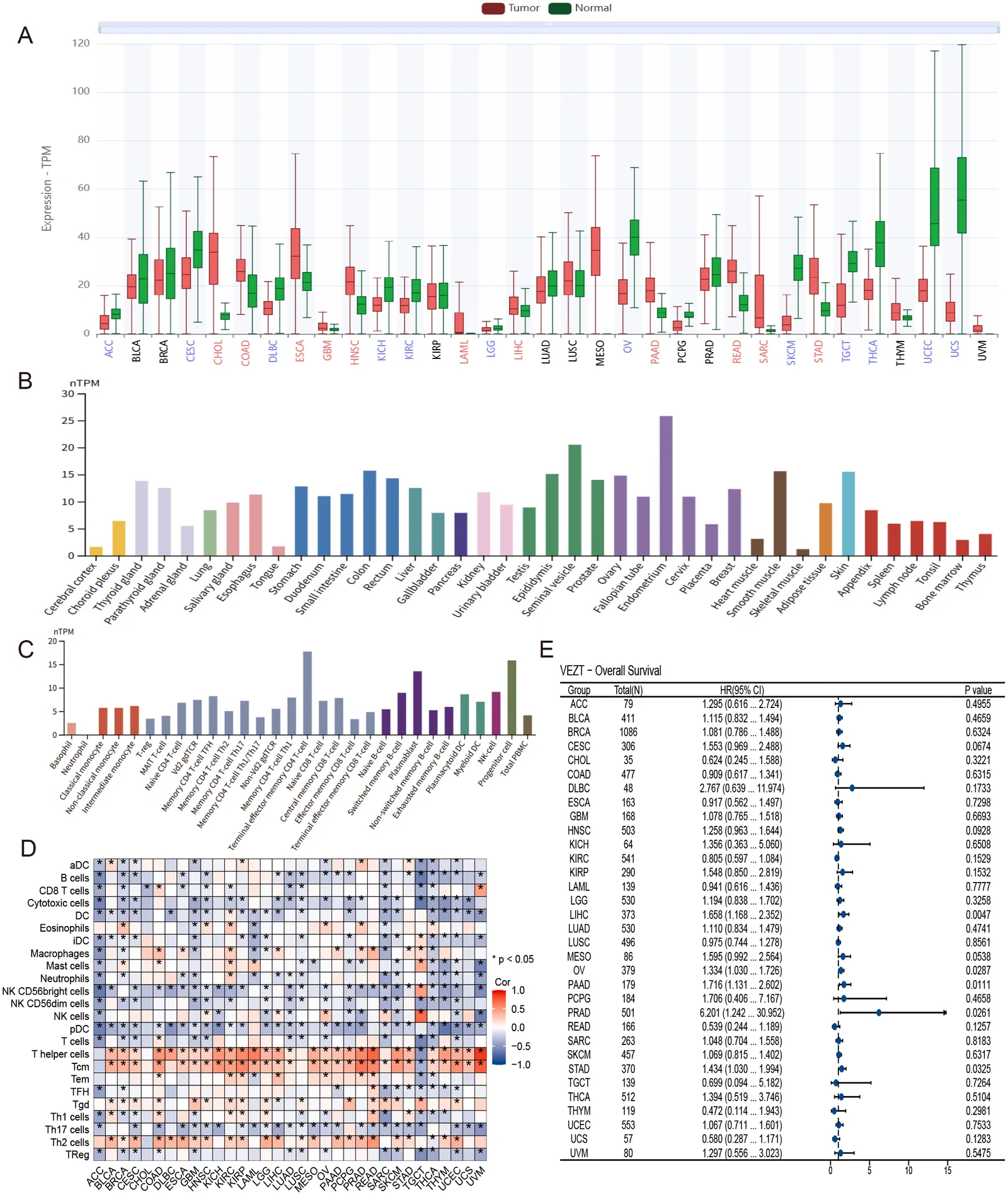
Expression characteristics of VEZT in pan-cancer and its association with immune infiltration and prognosis. **(A)** Differential expression of VEZT across 33 tumor types and corresponding normal tissues based on GEPIA2 (TCGA + GTEx datasets). **(B, C)** Expression distribution of VEZT in normal human tissues and at the single-cell level based on the Human Protein Atlas (HPA) database. **(D)** Correlation analysis between VEZT expression and immune cell infiltration levels across multiple cancers. **(E)** Cox regression analysis of the association between VEZT expression and overall survival (OS) in pan-cancer cohorts.

To characterize the expression profile of VEZT across human malignancies, we first performed a pan-cancer analysis using the GEPIA2 platform by integrating transcriptomic data from the TCGA and GTEx databases. As shown in **Figure 1A**, VEZT expression was significantly upregulated in multiple cancer types compared with corresponding normal tissues, particularly in pancreatic adenocarcinoma (PAAD), breast invasive carcinoma (BRCA), cholangiocarcinoma (CHOL), liver hepatocellular carcinoma (LIHC), lung adenocarcinoma (LUAD), and lung squamous cell carcinoma (LUSC).

To further investigate the physiological expression pattern of VEZT, data from the Human Protein Atlas (HPA) database were analyzed. The results demonstrated relatively stable VEZT protein expression across different normal tissues and cell types without marked tissue specificity (**Figures 1B, C**), suggesting that the aberrant upregulation of VEZT may be a tumor-associated event.

Subsequently, the relationship between VEZT expression and immune cell infiltration was evaluated using R software (ggplot2 v3.4.4). Correlation analysis revealed that VEZT expression was significantly associated with immune infiltration levels in multiple tumor types (**Figure 1D**), indicating a potential role of VEZT in regulating the tumor immune microenvironment. Furthermore, Cox proportional hazards regression analysis based on public datasets demonstrated that VEZT expression was significantly associated with patient prognosis in various cancer types (**Figure 1E**), suggesting its potential value as a prognostic biomarker.

### 3.2 Expression characteristics and clinical significance of VEZT in pancreatic cancer

**Figure 2:**
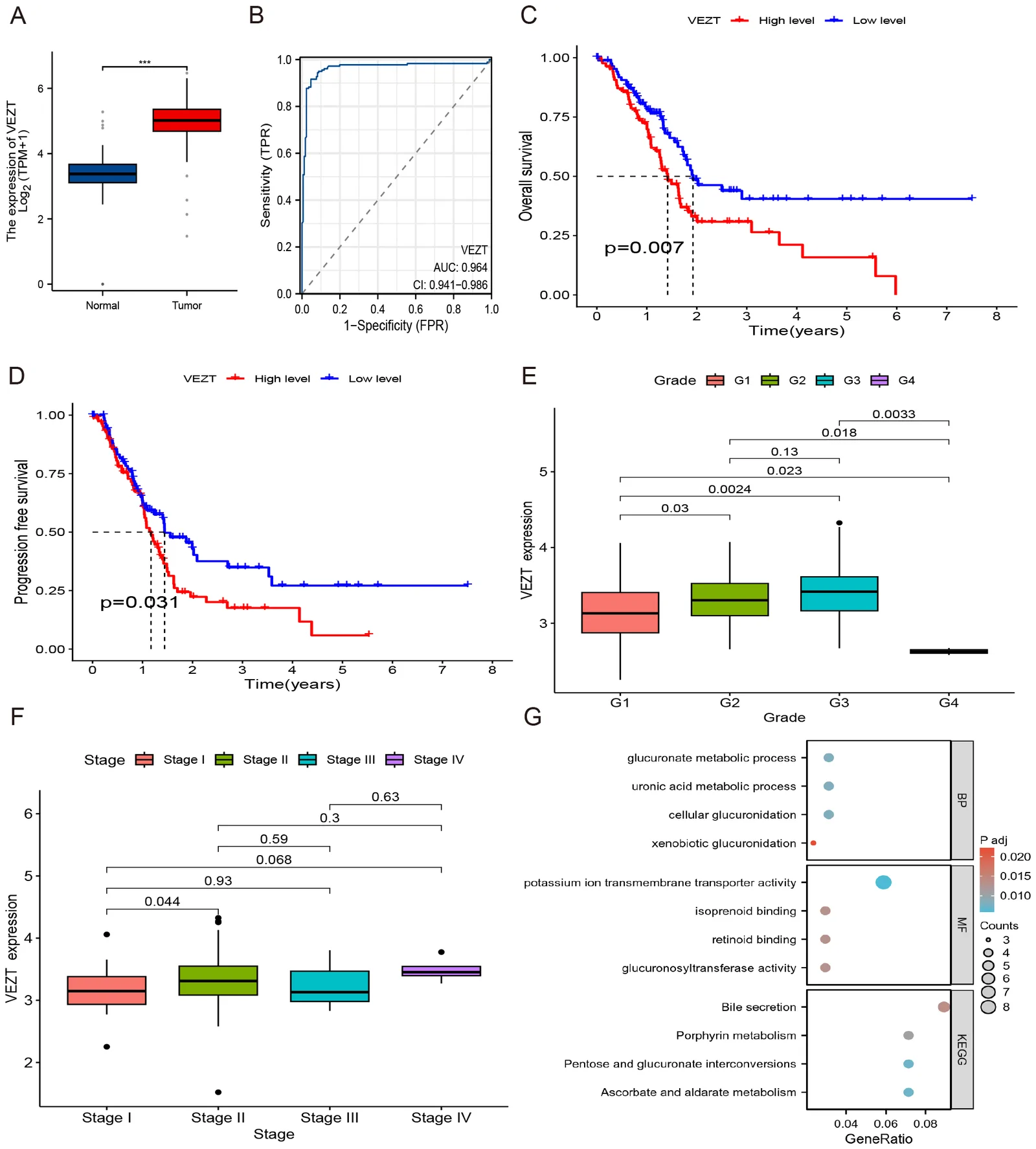
Expression characteristics and clinical significance of VEZT in pancreatic cancer. **(A)** VEZT expression levels in pancreatic cancer based on TCGA and GTEx datasets. **(B)** ROC curve analysis evaluating the diagnostic performance of VEZT in pancreatic cancer. **(C, D)** Kaplan–Meier survival analysis of overall survival (OS) and progression-free survival (PFS) stratified by VEZT expression. **(E, F)** Association of VEZT expression with tumor grade and clinical stage. **(G)** GO and KEGG enrichment analysis of VEZT-related genes.

Analysis of TCGA and GTEx datasets demonstrated that VEZT was significantly upregulated in pancreatic cancer tissues compared with normal pancreatic tissues (**Figure 2A**), which was consistent with the pan-cancer analysis and subsequent validation using clinical specimens. Receiver operating characteristic (ROC) curve analysis indicated that VEZT exhibited strong diagnostic and predictive performance in pancreatic cancer, as evidenced by a relatively high area under the curve (AUC) value (**Figure 2B**).

Kaplan–Meier survival analysis and univariate Cox regression analysis further demonstrated that elevated VEZT expression was significantly associated with shorter overall survival (OS) and progression-free survival (PFS) in patients with pancreatic cancer (**Figures 2C, D**), indicating that VEZT may serve as a potential prognostic biomarker. In addition, VEZT expression differed significantly among tumors with different histological grades (G1–G4) and pathological stages (stages I–II) (**Figures 2E, F**), suggesting that increased VEZT expression may be closely associated with pancreatic cancer progression.

### 3.3 GO and KEGG enrichment analyses of VEZT-associated genes

To explore the biological functions of VEZT in pancreatic cancer, Gene Ontology (GO) and Kyoto Encyclopedia of Genes and Genomes (KEGG) enrichment analyses were performed using the clusterProfiler package, with thresholds of P < 0.05 and FDR < 0.05. The results revealed that VEZT-associated genes were primarily enriched in glucuronate metabolic processes, glucuronidation, xenobiotic metabolic processes, and potassium ion transmembrane transport. These findings suggest that VEZT may participate in the regulation of cellular metabolic homeostasis and transmembrane transport activities.

KEGG pathway analysis further demonstrated significant enrichment in pathways related to bile secretion, porphyrin metabolism, pentose and glucuronate interconversions, and ascorbate and aldarate metabolism (**Figure 2G**). These pathways are closely associated with redox homeostasis, detoxification processes, and cellular energy metabolism, indicating that VEZT-associated genes may contribute to pancreatic cancer development through modulation of metabolic and oxidative stress-related pathways.

### 3.4 SLC25A24 was identified as a potential downstream target of VEZT

**Figure 3:**
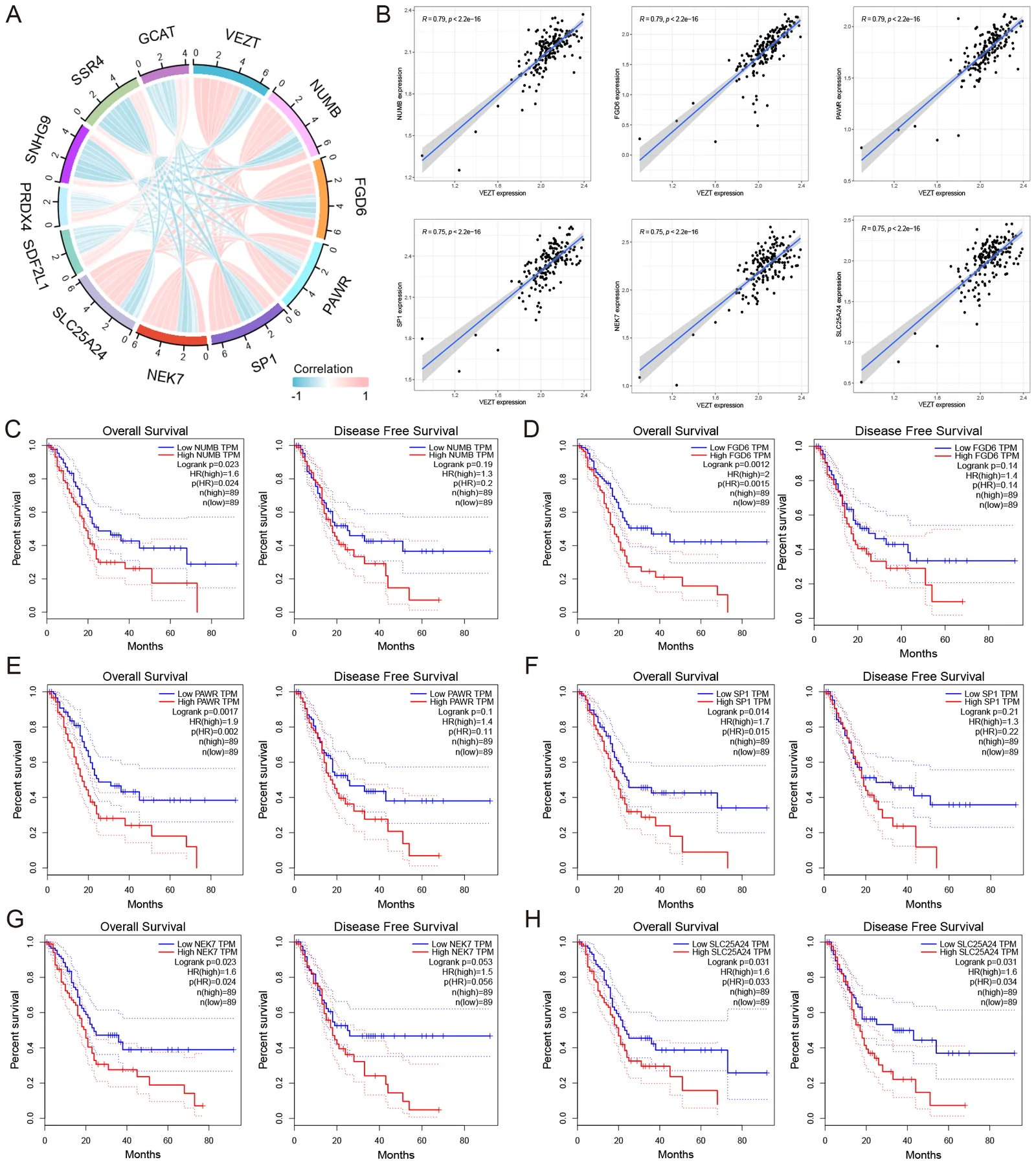
Identification of SLC25A24 as a downstream target of VEZT and functional analysis. **(A, B)** Screening of VEZT-related genes based on correlation analysis, identifying MUMB, FGD6, PAWR, SP1, NEK7, and SLC25A24 as significantly correlated genes. **(C–H)** Survival analysis of candidate genes in relation to overall survival (OS) and disease-free survival (DFS) in pancreatic cancer using the GEPIA database.

Correlation analysis identified several genes that were strongly associated with VEZT expression, including MUMB, FGD6, PAWR, SP1, NEK7, and SLC25A24 (**Figures 3A, B**). To identify potential downstream effectors of VEZT, we further evaluated the prognostic significance of these candidate genes using the GEPIA database. Kaplan–Meier survival analyses demonstrated that elevated SLC25A24 expression was significantly associated with poor overall survival (OS) and disease-free survival (DFS) in patients with pancreatic cancer (**Figures 3C–H**). Based on its strong correlation with VEZT and significant prognostic relevance, SLC25A24 was selected as the primary candidate for subsequent mechanistic investigations.

### 3.5 Expression of SLC25A24 in pancreatic cancer and its association with clinicopathological characteristics and immune correlation

**Figure 4:**
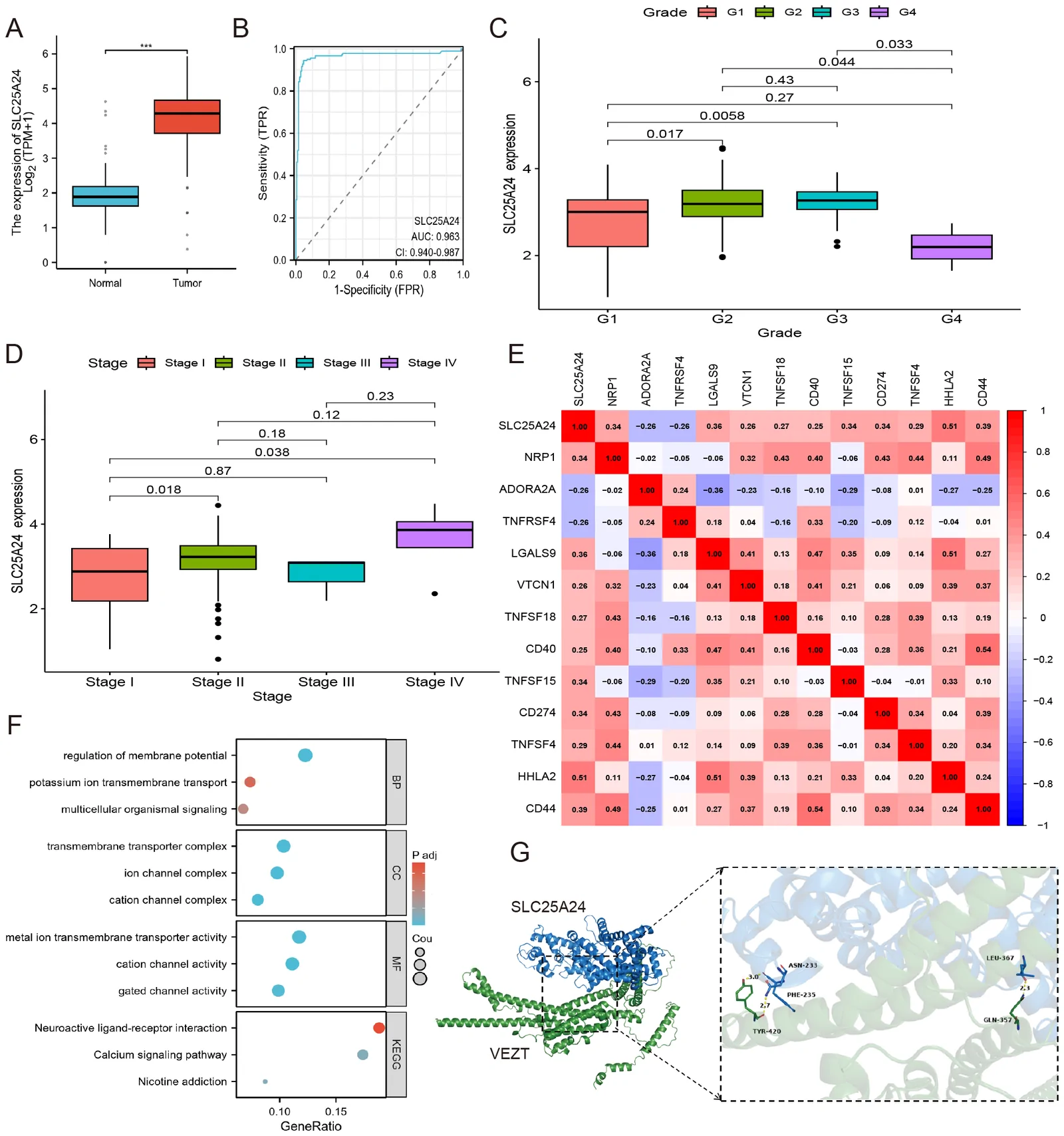
Expression, clinicopathological significance, and immune relevance of SLC25A24 in pancreatic cancer. **(A, B)** Expression level and ROC curve analysis of SLC25A24 in pancreatic cancer. **(C, D)** Association of SLC25A24 expression with tumor grade and clinical stage. **(E)** Correlation analysis between SLC25A24 and immune checkpoint genes. **(F)** GO and KEGG enrichment analysis of SLC25A24-related genes. **(G)** Molecular docking analysis between VEZT and SLC25A24 proteins.

To evaluate the expression pattern of SLC25A24 in pancreatic cancer, transcriptomic data from the GTEx and TCGA databases were analyzed. The results demonstrated that SLC25A24 was significantly upregulated in pancreatic cancer tissues compared with normal pancreatic tissues (**Figure 4A**). Receiver operating characteristic (ROC) curve analysis further revealed that SLC25A24 exhibited strong diagnostic and predictive performance in pancreatic cancer (**Figure 4B**). Subsequently, the association between SLC25A24 expression and clinicopathological characteristics was investigated. Wilcoxon rank-sum analysis showed that SLC25A24 expression was significantly associated with tumor grade (G1–G4) and pathological stage (stages I–IV) (**Figures 4C, D**), suggesting that elevated SLC25A24 expression may contribute to pancreatic cancer progression.

To further explore the relationship between SLC25A24 and the tumor immune microenvironment, Pearson correlation analysis was performed to evaluate the association between SLC25A24 expression and immune checkpoint-related genes. The results demonstrated that SLC25A24 expression was negatively correlated with several immune checkpoint genes, including ADORA2A and TNFRSF4 (**Figure 4E**), indicating that SLC25A24 may participate in immune regulation and remodeling of the pancreatic cancer immune microenvironment.

### 3.6 GO and KEGG enrichment analyses of SLC25A24-associated genes and molecular docking analysis between VEZT and SLC25A24

To investigate the potential biological functions of SLC25A24 in pancreatic cancer, GO and KEGG enrichment analyses were conducted using the clusterProfiler package in R. The results demonstrated that SLC25A24-associated genes were primarily enriched in biological processes related to membrane potential regulation, potassium ion transmembrane transport, metal ion transmembrane transporter activity, cation channel activity, and gated channel activity. KEGG pathway analysis further revealed significant enrichment in pathways including Neuroactive ligand–receptor interaction, Calcium signaling pathway, and Nicotine addiction (**Figure 4F**). These findings suggest that SLC25A24-associated genes are mainly involved in the maintenance of ion homeostasis and calcium signaling pathways, thereby contributing to pancreatic cancer development and progression.

To further investigate the potential interaction between VEZT and SLC25A24, molecular docking analysis was performed using the HDOCK online platform, followed by structural visualization with PyMOL. The results revealed a stable binding interface between VEZT and SLC25A24 (**Figure 4G**), suggesting that VEZT may regulate SLC25A24 function through direct protein–protein interaction.

### 3.7 Diagnostic and prognostic performance of the combined VEZT/SLC25A24 model

**Figure 5:**
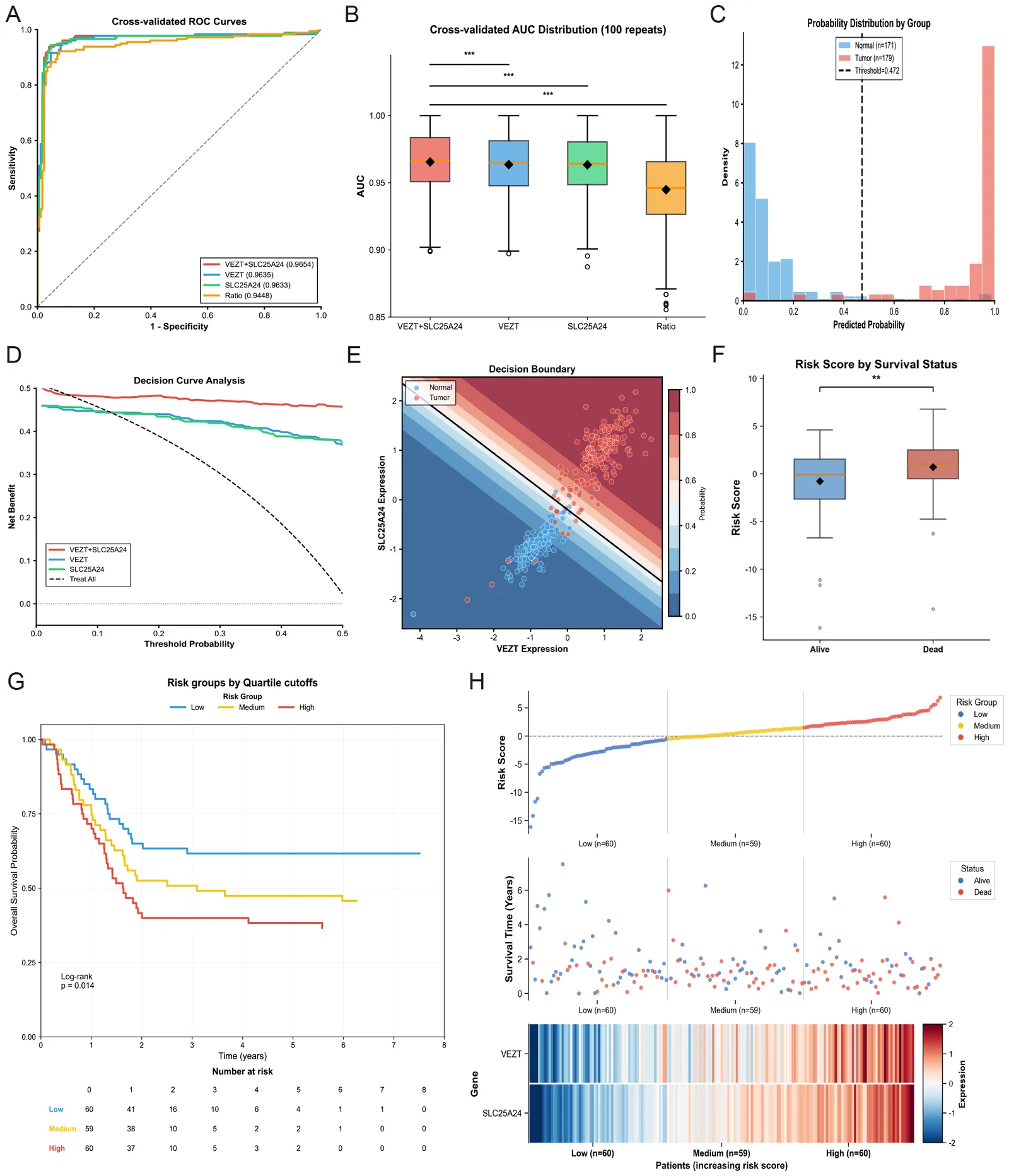
Construction and evaluation of a combined diagnostic and prognostic model based on VEZT and SLC25A24. **(A, B)** ROC curve analysis of VEZT, SLC25A24, and the combined model. **(C)** Risk distribution based on the optimal cutoff value determined by the Youden index. **(D)** Decision curve analysis (DCA) evaluating the clinical utility of the combined model. **(E)** Two-dimensional scatter plot showing classification performance of the model for tumor and normal samples. **(F)** Comparison of risk scores between deceased and surviving patients. **(G)** Kaplan–Meier survival analysis stratified by risk score. **(H)** Heatmap of VEZT and SLC25A24 expression across risk groups.

To improve the diagnostic performance for pancreatic cancer, a logistic regression model integrating VEZT and SLC25A24 expression was constructed and evaluated using 100 rounds of repeated five-fold cross-validation. ROC curve analysis demonstrated that the combined model achieved an AUC of 0.9654, which was higher than that of VEZT alone (AUC = 0.9635), SLC25A24 alone (AUC = 0.9633), and the gene-ratio model (AUC = 0.09448), indicating superior diagnostic performance of the dual-gene model (**Figures 5A, B**).

The optimal cutoff value determined by the Youden index was 0.472. Minimal overlap was observed between tumor and normal samples around this threshold, indicating excellent discrimination capability (**Figure 5C**). The risk score formula was defined as follows: Risk Score = −9.902200 + 1.067189 × VEZT + 1.899521 × SLC25A24.

Decision curve analysis (DCA) demonstrated that the combined model achieved the highest net clinical benefit across most threshold probability ranges, highlighting its potential clinical utility (**Figure 5D**). A two-dimensional scatter plot illustrated the decision boundary generated by the expression levels of VEZT and SLC25A24. Normal samples with relatively low expression levels clustered in the lower-left region, whereas tumor samples with elevated expression levels were predominantly distributed in the upper-right region, indicating that combined detection effectively distinguished tumor samples from normal controls (**Figure 5E**).

Prognostic analysis revealed that deceased patients exhibited significantly higher risk scores than surviving patients (4.15 ± 2.84 vs. 3.19 ± 2.97, t = 2.99, P = 0.003) (**Figure 5F**). Kaplan–Meier survival analysis based on risk-score tertiles demonstrated a progressive increase in mortality risk with increasing risk scores (**Figure 5G**). Furthermore, the heatmap integrating risk scores, survival status, survival time, and VEZT/SLC25A24 expression levels showed that both genes were markedly upregulated in the high-risk group and were closely associated with unfavorable prognosis (**Figure 5H**). Collectively, these findings indicate that the combined VEZT/SLC25A24 model exhibits excellent performance in both diagnosis and prognostic prediction of pancreatic cancer and may possess considerable clinical application value.

### 3.8 Single-cell and spatial transcriptomic analyses of VEZT and SLC25A24

**Figure 6:**
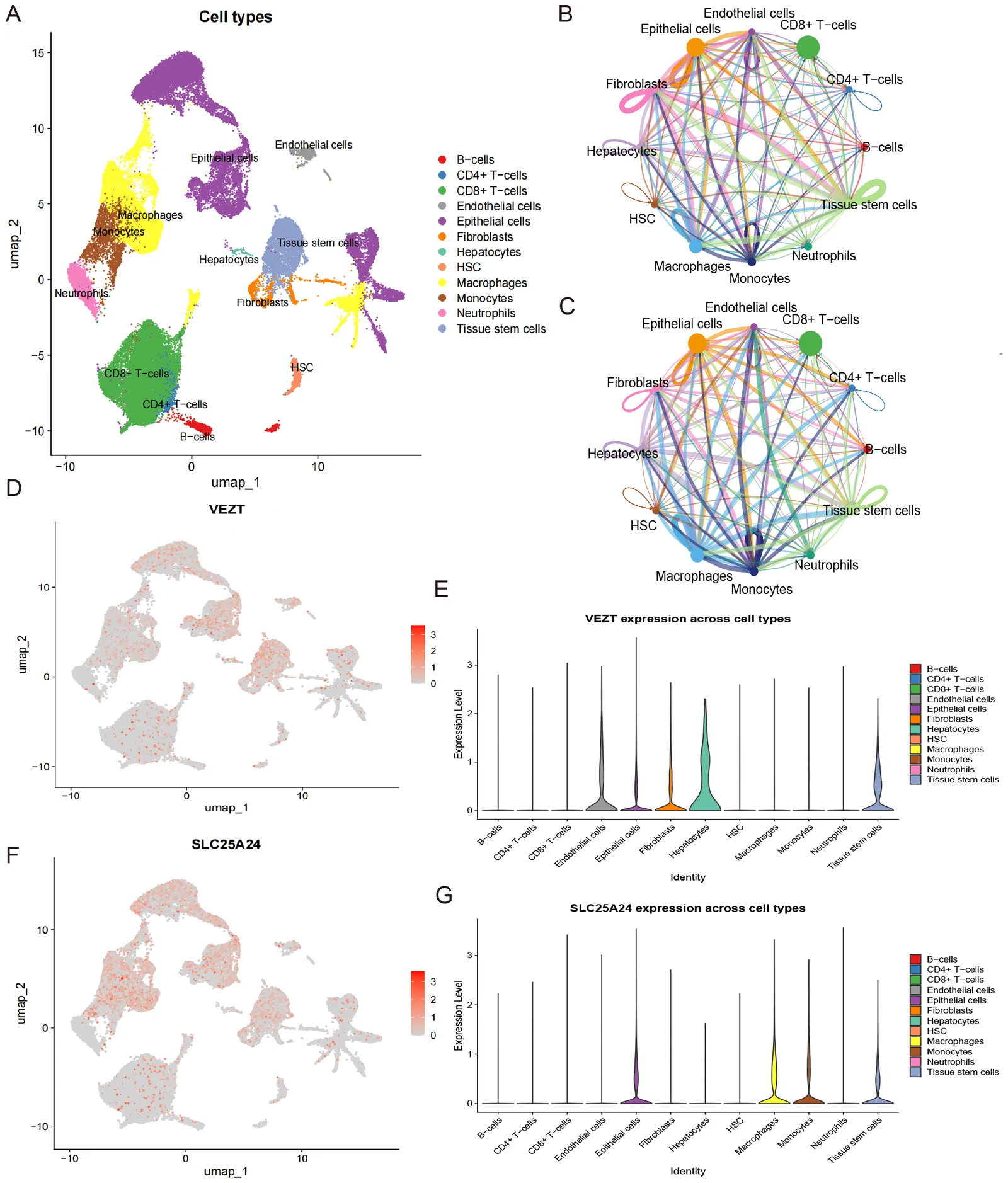
Single-cell analysis of VEZT and SLC25A24. **(A)** UMAP visualization identifying 12 major cell types. **(B, C)** Cell–cell communication network analysis within the tumor microenvironment. **(D, E)** Expression distribution of VEZT across different cell types. **(F, G)** Expression distribution of SLC25A24 across different cell types.

To further characterize the cellular distribution and expression patterns of VEZT and SLC25A24 within the tumor microenvironment, single-cell RNA sequencing analyses were performed. Based on UMAP dimensionality reduction and clustering analysis, twelve major cell populations were identified, including B cells, CD4^+^ T cells, CD8^+^ T cells, NK cells, macrophages, monocytes, neutrophils, epithelial cells, hepatocytes, fibroblasts, endothelial cells, and stem cell-related populations (hematopoietic stem cells and tissue stem cells) (**Figure 6A**).

Cell–cell communication analysis revealed extensive ligand–receptor interactions among epithelial cells, endothelial cells, T cells, and fibroblasts, forming a major communication network within the pancreatic cancer microenvironment (**Figures 6B, C**). Further analysis demonstrated that VEZT was highly expressed in endothelial cells and epithelial cells (**Figures 6D, E**). Similarly, SLC25A24 expression was predominantly enriched in epithelial cells (**Figures 6F, G**).

### 3.9 Independent prognostic significance and immune infiltration correlation analyses of VEZT and SLC25A24

**Figure 7:**
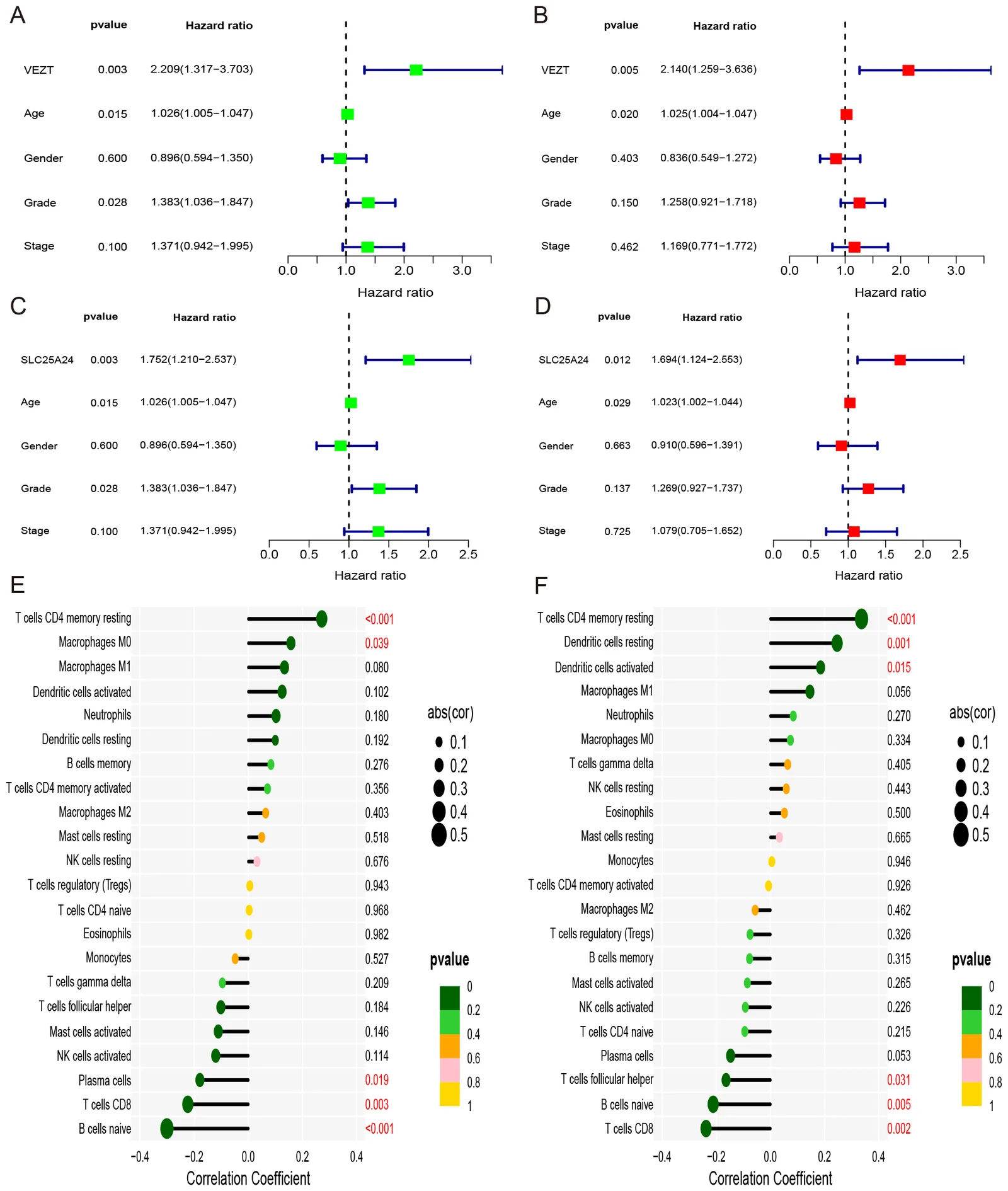
Independent prognostic value and immune infiltration association of VEZT and SLC25A24. **(A–D)** Univariate and multivariate Cox regression analyses assessing the independent prognostic value of VEZT and SLC25A24. **(E, F)** Correlation analysis between the two genes and immune cell infiltration using the CIBERSORT algorithm.

To further evaluate the prognostic significance of VEZT and SLC25A24 in pancreatic cancer, univariate and multivariate Cox regression analyses were performed. The results demonstrated that, after adjustment for clinicopathological variables, both VEZT and SLC25A24 remained significantly associated with patient prognosis and were identified as independent prognostic factors for pancreatic cancer (**Figures 7A–D**).In addition, the CIBERSORT algorithm was employed to evaluate the associations between gene expression and the infiltration levels of 22 immune cell populations. The results revealed highly similar immune infiltration patterns for VEZT and SLC25A24 (**Figures 7E, F**). Specifically, both genes exhibited significant positive correlations with resting memory CD4^+^ T-cell infiltration, whereas significant negative correlations were observed with CD8^+^ T cells and naïve B cells. Furthermore, VEZT expression was significantly associated with infiltration of M0 macrophages and plasma cells, whereas SLC25A24 expression was significantly correlated with both resting and activated dendritic cells.

These findings indicate that VEZT and SLC25A24 are closely associated with immune cell infiltration in pancreatic cancer and exhibit remarkably similar immune-related characteristics. Combined with their prognostic significance, these results suggest that VEZT and SLC25A24 may participate in the regulation of the pancreatic cancer immune microenvironment and contribute to tumor progression.

### 3.10 VEZT is highly expressed in pancreatic cancer tissues and cell lines

**Figure 8:**
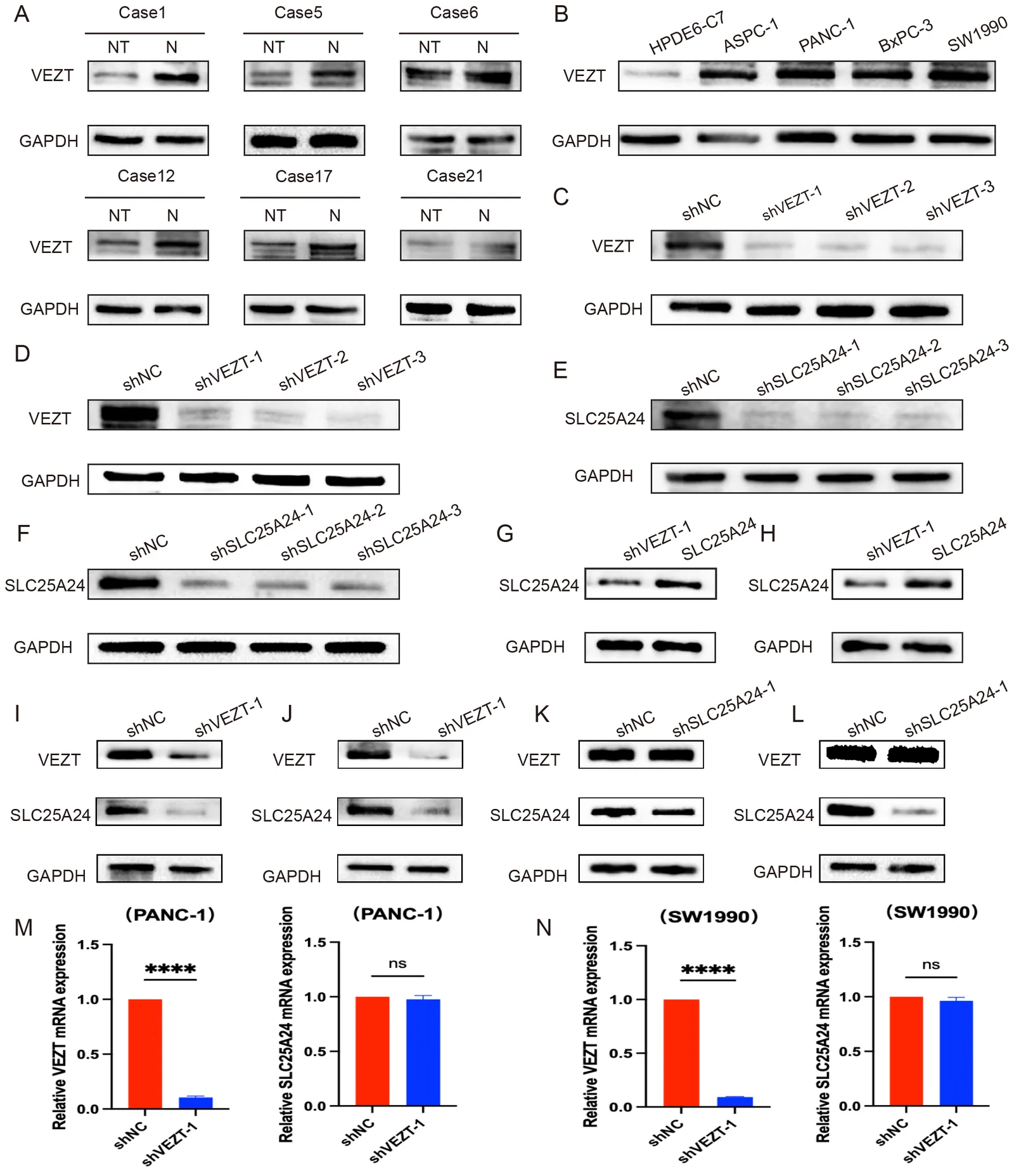
Expression validation and establishment of stable cell models for VEZT and SLC25A24. **(A)** VEZT protein expression in pancreatic cancer tissues and adjacent normal tissues. **(B)** VEZT expression in pancreatic cancer cell lines and normal pancreatic epithelial cells. **(C)** Validation of VEZT knockdown efficiency in PANC-1 cells. **(D)** Validation of VEZT knockdown efficiency in SW1990 cells. **(E)** Validation of SLC25A24 knockdown efficiency in PANC-1 cells. **(F)** Validation of SLC25A24 knockdown efficiency in SW1990 cells. **(G, H)** Establishment of SLC25A24 rescue overexpression models in PANC-1 and SW1990 cells. **(I, J)** Effect of VEZT knockdown on SLC25A24 protein expression in PANC-1 and SW1990 cells. **(K, L)** Effect of SLC25A24 knockdown on VEZT protein expression in PANC-1 and SW1990 cells. **(M, N)** Changes in SLC25A24 mRNA expression following VEZT knockdown in PANC-1 and SW1990 cells. ****P < 0.0001.

To validate the bioinformatics findings, six pairs of pancreatic cancer tissues and matched adjacent non-tumorous tissues were randomly selected for Western blot analysis of VEZT protein expression. The results showed that VEZT protein levels were markedly elevated in pancreatic cancer tissues compared with adjacent normal tissues (**Figure 8A**), which was consistent with the results obtained from public database analyses.

Subsequently, VEZT protein expression was examined in the normal pancreatic ductal epithelial cell line HPDE6-C7 and pancreatic cancer cell lines PANC-1, SW1990, AsPC-1, and BxPC-3. Western blot analysis demonstrated that VEZT expression was significantly higher in all pancreatic cancer cell lines than in HPDE6-C7 cells (**Figure 8B**). Based on their relatively high VEZT expression levels, PANC-1 and SW1990 cells were selected for subsequent functional studies.

### 3.11 Construction of stable VEZT and SLC25A24 knockdown cell models and analysis of their regulatory relationship

To investigate the biological functions of VEZT and SLC25A24 in pancreatic cancer, stable knockdown cell models were established in PANC-1 and SW1990 cells. Western blot analysis confirmed that the protein expression levels of VEZT and SLC25A24 were significantly reduced following lentiviral transduction (**Figures 8C–F**), indicating successful establishment of the stable knockdown models. To further investigate the functional relationship between VEZT and SLC25A24, a rescue model was generated by restoring SLC25A24 expression in VEZT-knockdown cells. Western blot analysis confirmed successful restoration of SLC25A24 protein expression (**Figures 8G, H**).

To clarify the regulatory relationship between the two proteins, their expression levels were subsequently examined. VEZT knockdown markedly reduced SLC25A24 protein expression in both PANC-1 and SW1990 cells (**Figures 8I, J**). In contrast, knockdown of SLC25A24 did not significantly affect VEZT protein expression (**Figures 8K, L**), suggesting that VEZT may function upstream of SLC25A24 and positively regulate its expression.

Furthermore, quantitative real-time PCR analysis revealed that VEZT knockdown did not significantly alter SLC25A24 mRNA expression levels (**Figures 8M, N**), indicating that VEZT-mediated regulation of SLC25A24 is unlikely to occur at the transcriptional level and may instead involve post-translational regulation or modulation of protein stability.

### 3.12 VEZT promotes the proliferation and migration of pancreatic cancer cells

**Figure 9:**
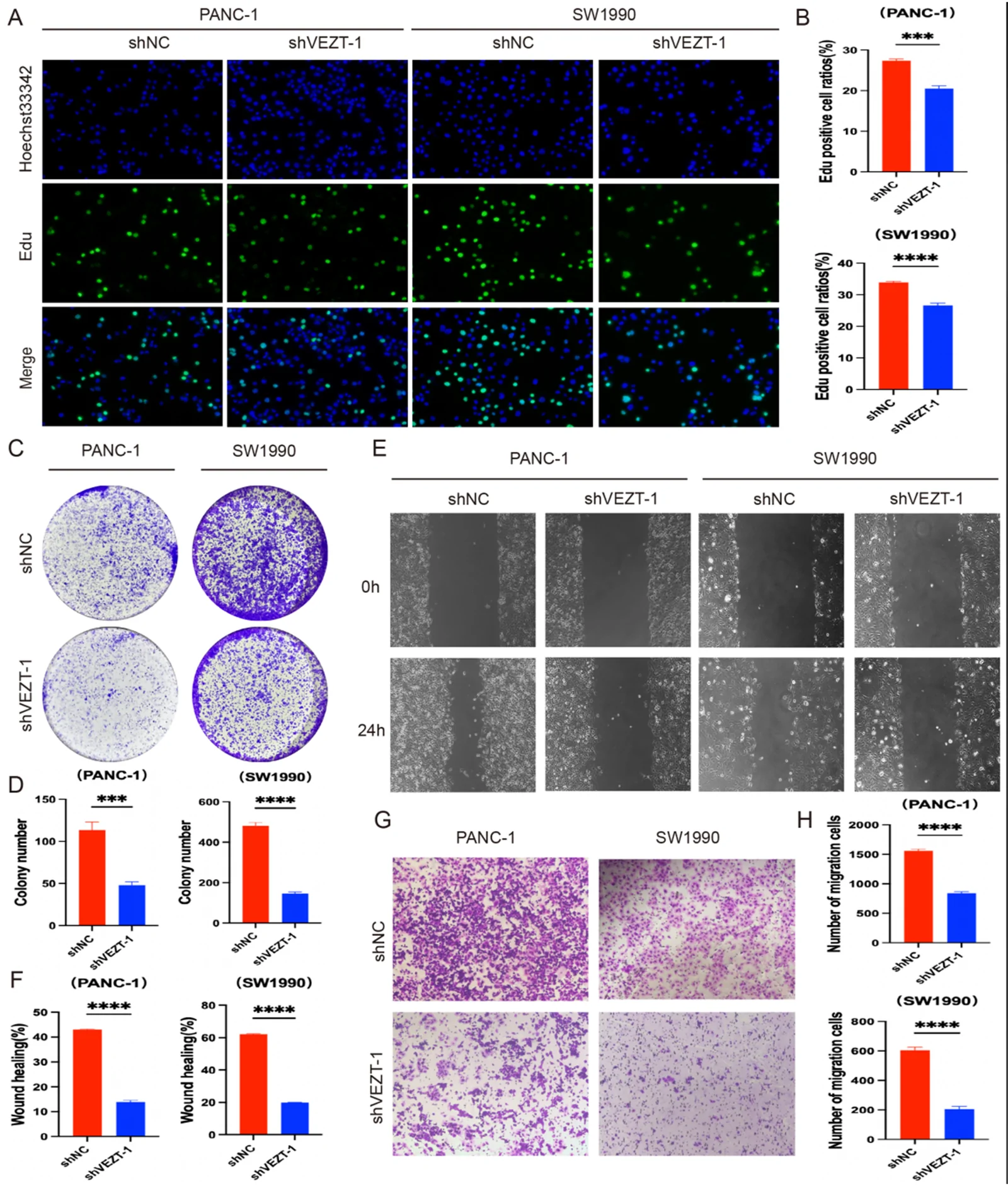
VEZT promotes proliferation and migration of pancreatic cancer cells. **(A–D)** EdU incorporation and colony formation assays assessing the effect of VEZT on cell proliferation. **(E–H)** Wound healing and Transwell assays evaluating the effect of VEZT on cell migration. ***P < 0.001, and ****P < 0.0001.

To evaluate the role of VEZT in pancreatic cancer progression, EdU incorporation and colony formation assays were performed to assess cellular proliferative capacity. The results demonstrated that VEZT knockdown significantly inhibited the proliferation of both PANC-1 and SW1990 cells (**Figures 9A–D**). Cell migration was subsequently evaluated using Transwell migration and wound-healing assays. VEZT silencing markedly impaired the migratory ability of pancreatic cancer cells compared with control cells (**Figures 9E–H**). These findings indicate that VEZT promotes the proliferation and migration of pancreatic cancer cells.

### 3.13 SLC25A24 promotes the proliferation and migration of pancreatic cancer cells

**Figure 10:**
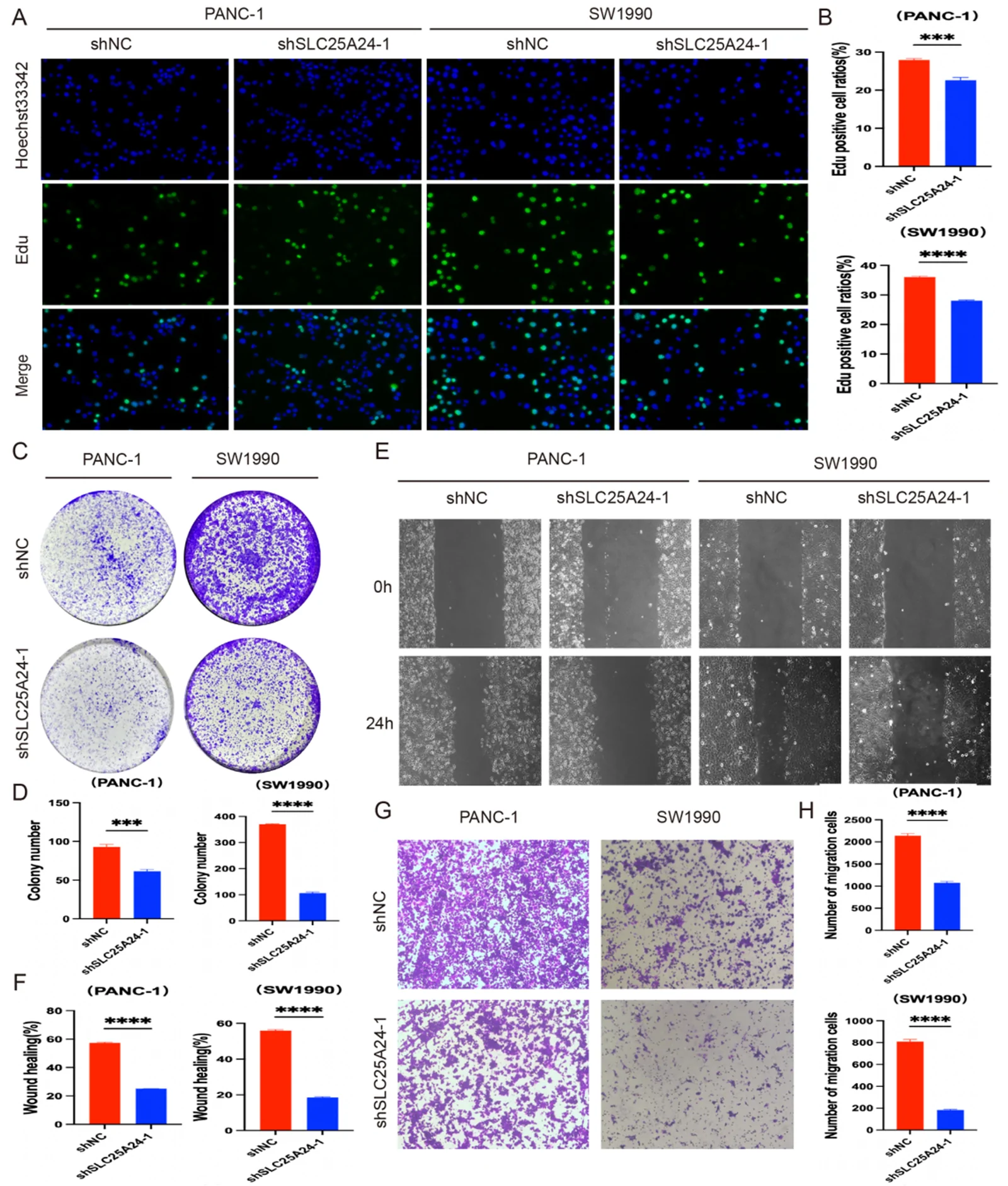
SLC25A24 promotes proliferation and migration of pancreatic cancer cells. **(A–D)** EdU incorporation and colony formation assays assessing the effect of SLC25A24 on cell proliferation. **(E–H)** Wound healing and Transwell assays evaluating the effect of SLC25A24 on cell migration. ***P < 0.001, and ****P < 0.0001.

To determine the biological role of SLC25A24 in pancreatic cancer, EdU incorporation and colony formation assays were conducted following SLC25A24 knockdown. The results demonstrated that silencing SLC25A24 significantly suppressed pancreatic cancer cell proliferation (**Figures 10A–D**). Similarly, Transwell migration and wound-healing assays revealed that SLC25A24 knockdown markedly reduced the migratory capacity of pancreatic cancer cells (**Figures 10E–H**). These results indicate that SLC25A24 exerts a tumor-promoting role and contributes to the malignant progression of pancreatic cancer.

### 3.14 Restoration of SLC25A24 expression reverses the suppressive effects induced by VEZT knockdown

**Figure 11:**
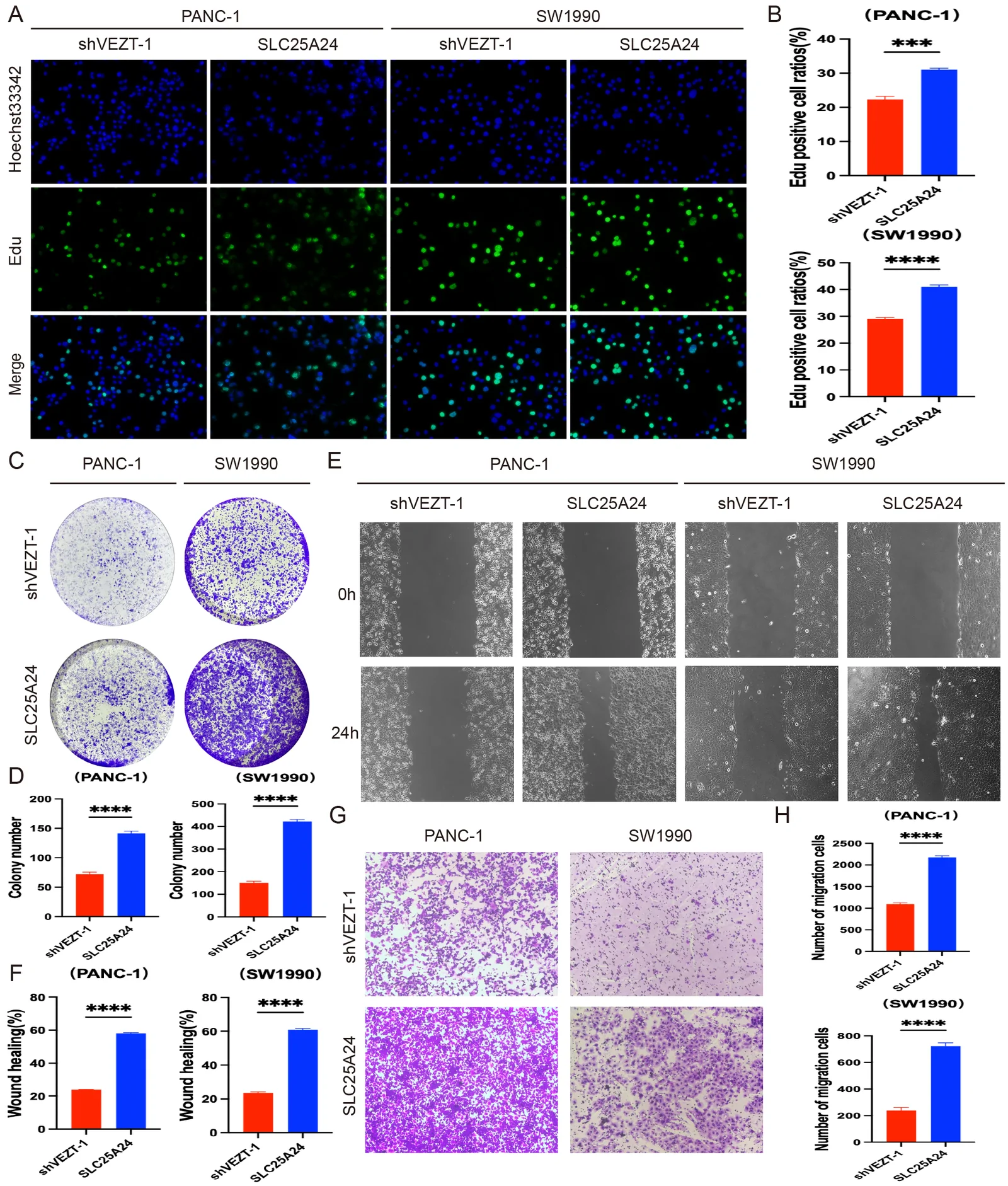
SLC25A24 rescue reverses the inhibitory effects induced by VEZT knockdown. **(A–D)** Rescue of VEZT knockdown-induced suppression of cell proliferation by SLC25A24 overexpression. **(E–H)** Rescue of VEZT knockdown-induced inhibition of cell migration by SLC25A24 overexpression. ***P < 0.001, and ****P < 0.0001.

To determine whether VEZT exerts its oncogenic functions through SLC25A24, rescue experiments were performed by restoring SLC25A24 expression in VEZT-knockdown cells.EdU incorporation and colony formation assays demonstrated that restoration of SLC25A24 expression significantly rescued the impaired proliferative capacity caused by VEZT depletion (**Figures 11A–D**). Consistently, Transwell migration and wound-healing assays showed that re-expression of SLC25A24 substantially restored the migratory ability of VEZT-deficient pancreatic cancer cells (**Figures 11E–H**). These findings suggest that the tumor-promoting effects of VEZT on pancreatic cancer cell proliferation and migration are at least partially dependent on SLC25A24.

### 3.15 VEZT interacts with SLC25A24 and maintains its protein stability

**Figure 12:**
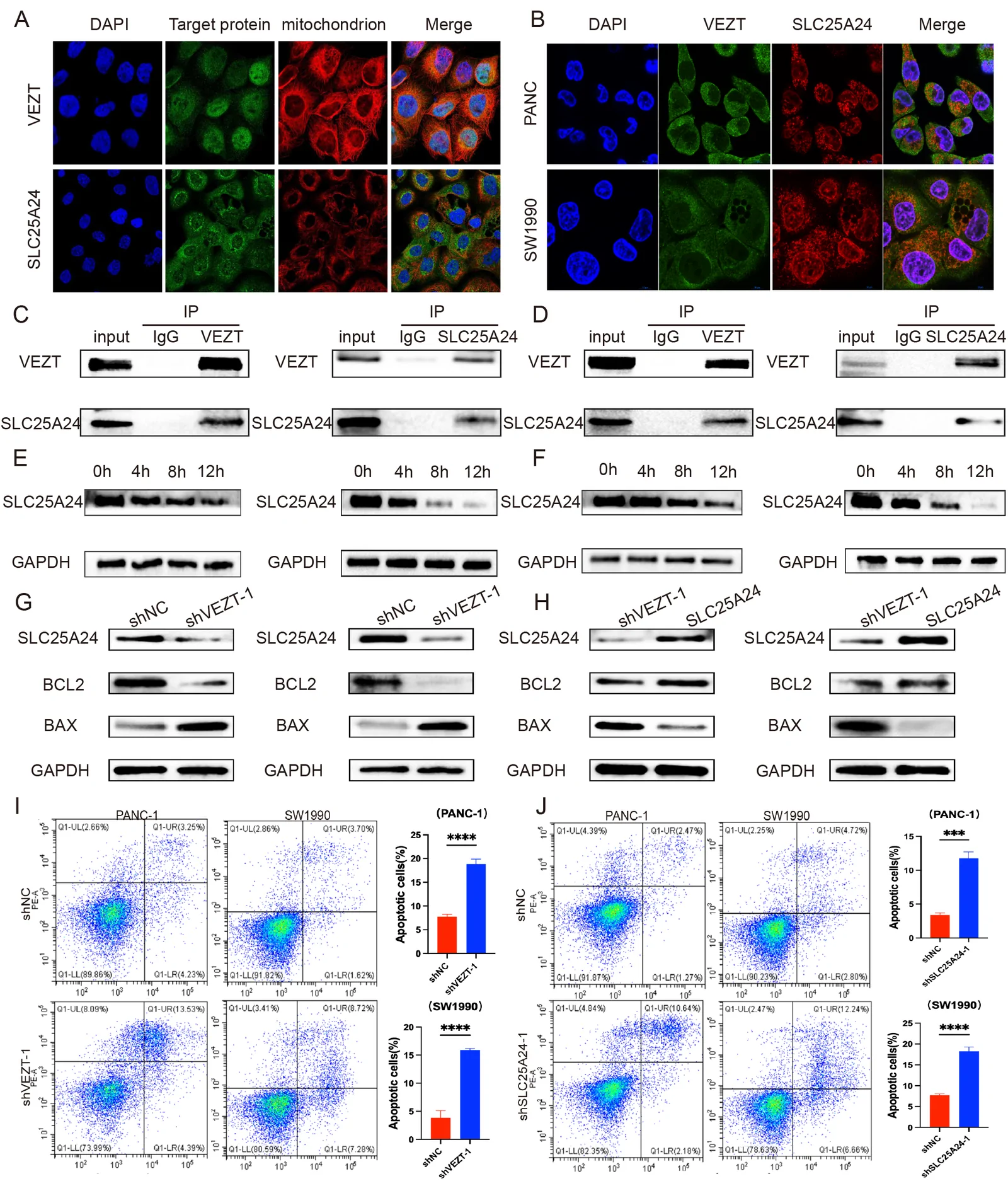
Interaction between VEZT and SLC25A24 and regulation of apoptosis. **(A)** Confocal immunofluorescence images of A-431 cells obtained from the Human Protein Atlas (HPA) database,. **(B)** Immunofluorescence colocalization analysis of VEZT and SLC25A24 in pancreatic cancer cells. **(C)** Co-immunoprecipitation (Co-IP) assays demonstrating interaction between VEZT and SLC25A24 in PANC-1 cells. **(D)** Co-immunoprecipitation (Co-IP) assays demonstrating interaction between VEZT and SLC25A24 in SW1990 cells. **(E)** CHX chase assays comparing SLC25A24 protein stability between control and VEZT knockdown groups in PANC-1 cells. **(F)** CHX chase assays assessing SLC25A24 protein stability following VEZT knockdown in SW1990 cells. **(G)** Effect of VEZT knockdown on BCL2 and BAX expression in PANC-1 and SW1990 cells. **(H)** Effect of SLC25A24 rescue on apoptosis-related protein expression. **(I, J)** Flow cytometry analysis of apoptosis levels. ***P < 0.001, and ****P < 0.0001.

To preliminarily assess the potential spatial association between VEZT and SLC25A24 within cells, we first analyzed their subcellular localization using data from the Human Protein Atlas (HPA) database. VEZT was predominantly distributed in the nucleoplasm and cytoplasm, whereas SLC25A24 was primarily localized to the mitochondria (**Figure 12A**), suggesting that the two proteins may have the potential to become spatially proximal within specific subcellular regions. Immunofluorescence colocalization analysis in pancreatic cancer cells subsequently revealed partial overlap between the fluorescence signals of VEZT and SLC25A24, further indicating a degree of intracellular spatial proximity between the two proteins (**Figure 12B**). On this basis, Co-IP assays showed that VEZT and SLC25A24 could be detected within the same protein complex (**Figures 12C, D**), further supporting a potential protein–protein association between them.

To further elucidate the mechanism underlying VEZT-mediated regulation of SLC25A24, cycloheximide (CHX) chase assays were conducted to evaluate SLC25A24 protein stability. Compared with control cells, VEZT knockdown significantly accelerated the degradation of SLC25A24 protein and markedly shortened its half-life (**Figures 12E, F**). These findings suggest that VEZT maintains SLC25A24 expression by directly interacting with and stabilizing the SLC25A24 protein.

### 3.16 VEZT regulates apoptosis of pancreatic cancer cells through SLC25A24

**Figure 13:**
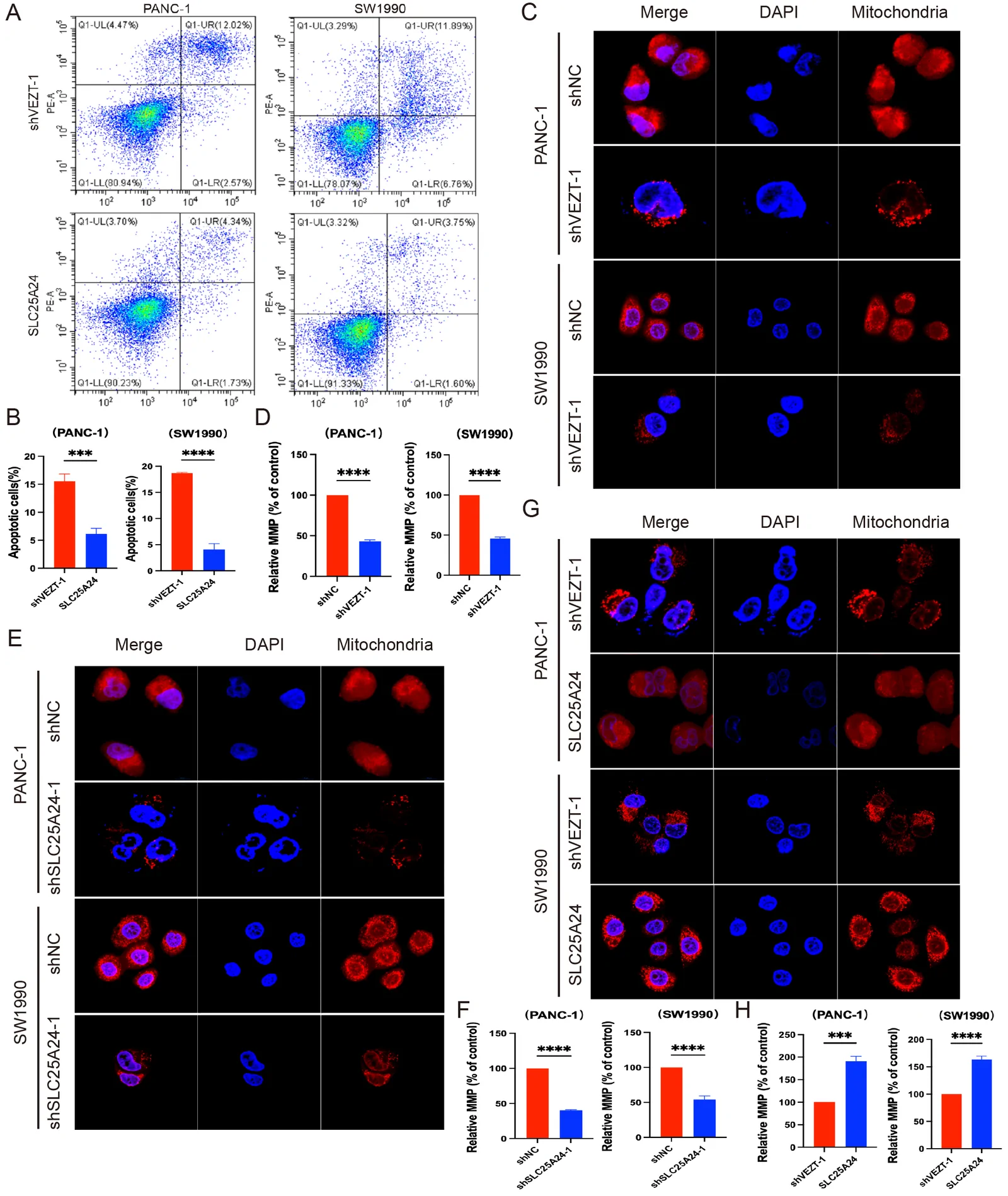
VEZT maintains mitochondrial homeostasis via SLC25A24. **(A, B)** Effect of SLC25A24 rescue on VEZT knockdown-induced apoptosis. **(C, D)** MitoTracker Red CMXRos staining showing mitochondrial structural disruption and reduced membrane potential following VEZT knockdown. **(E, F)** Mitochondrial dysfunction induced by SLC25A24 knockdown. **(G, H)** Restoration of mitochondrial integrity following SLC25A24 rescue in VEZT knockdown cells. ***P < 0.001, and ****P < 0.0001.

To investigate the role of the VEZT/SLC25A24 axis in apoptosis regulation, the expression levels of apoptosis-related proteins were examined. Western blot analysis showed that VEZT knockdown significantly reduced the expression of the anti-apoptotic protein BCL2 while markedly increasing the expression of the pro-apoptotic protein BAX (**Figure 12G**). Notably, restoration of SLC25A24 expression reversed these changes, leading to increased BCL2 expression and decreased BAX expression (**Figure 12H**). Flow cytometric analysis further demonstrated that silencing either VEZT or SLC25A24 significantly increased apoptotic cell populations in pancreatic cancer cells (**Figures 12I, J**). In contrast, restoration of SLC25A24 expression markedly attenuated apoptosis induced by VEZT knockdown (**Figures 13A, B**). Collectively, these results indicate that VEZT suppresses pancreatic cancer cell apoptosis by maintaining SLC25A24 expression.

### 3.17 VEZT maintains mitochondrial function through SLC25A24

To further evaluate the effect of the VEZT/SLC25A24 axis on mitochondrial membrane potential, MitoTracker Red CMXRos staining was performed. Compared with control cells, VEZT knockdown significantly reduced MitoTracker Red CMXRos fluorescence intensity, indicating a decrease in mitochondrial membrane potential (**Figures 13C, D**). Similarly, SLC25A24 depletion markedly decreased MitoTracker Red CMXRos fluorescence intensity, suggesting that loss of SLC25A24 impairs mitochondrial membrane potential (**Figures 13E, F**). Notably, restoration of SLC25A24 expression in VEZT-knockdown cells partially recovered MitoTracker Red CMXRos fluorescence intensity, indicating that SLC25A24 overexpression partially reverses the reduction in mitochondrial membrane potential induced by VEZT depletion (**Figures 13G, H**).

**Figure 14:**
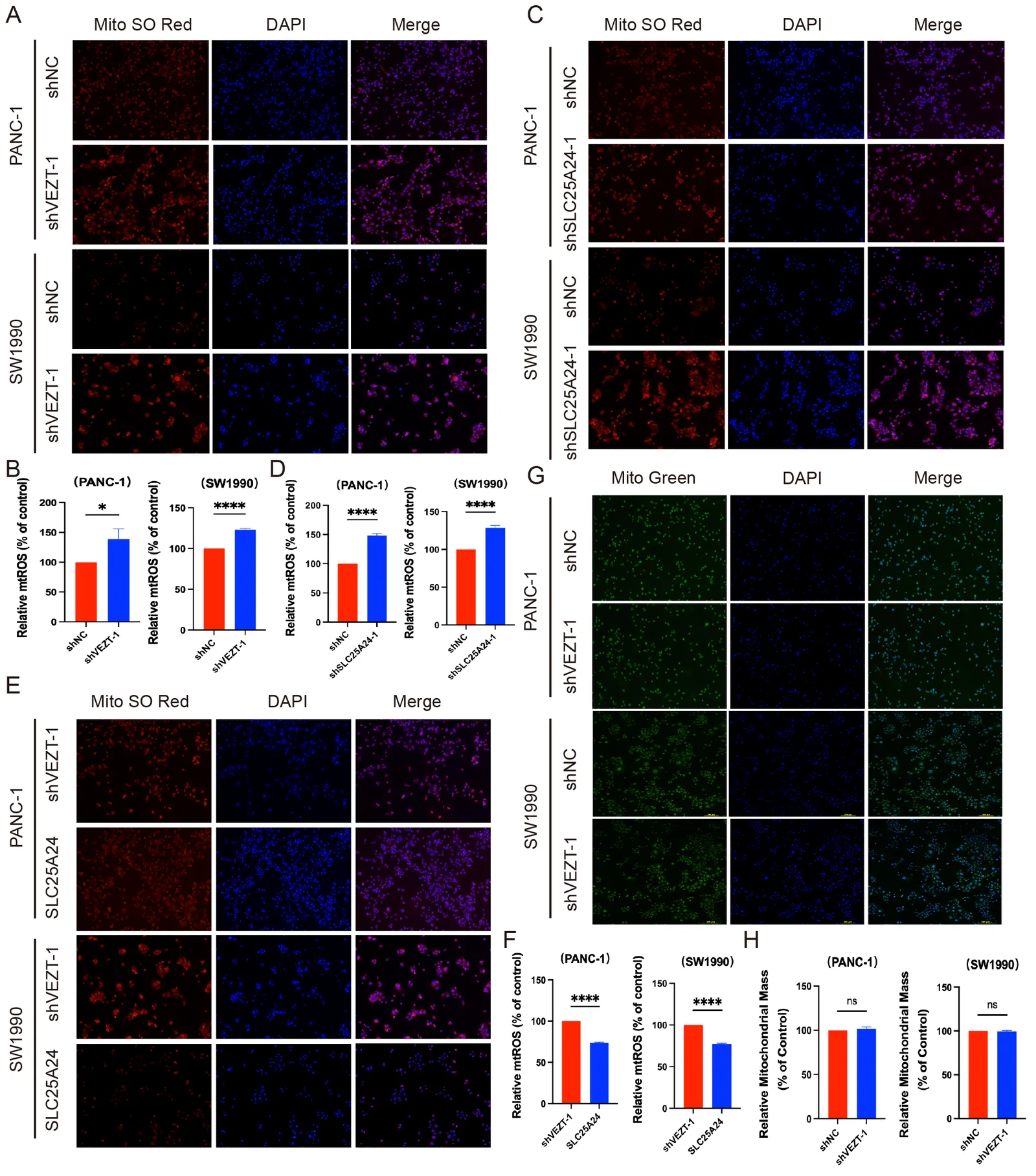
The VEZT/SLC25A24 axis regulates mitochondrial oxidative stress and mitochondrial mass in pancreatic cancer cells. **(A, B)** Representative MitoSOX Red fluorescence images and quantitative analysis in control and VEZT-knockdown pancreatic cancer cells, used to assess mitochondrial superoxide levels. **(C, D)** Representative MitoSOX Red fluorescence images and quantitative analysis in control and SLC25A24-knockdown pancreatic cancer cells, used to assess mitochondrial superoxide levels. **(E, F)** Representative MitoSOX Red fluorescence images and quantitative analysis before and after restoration of SLC25A24 expression in VEZT-knockdown cells, used to assess mitochondrial superoxide levels. **(G, H)** Representative MitoTracker Green FM fluorescence images and quantitative analysis in control and VEZT-knockdown pancreatic cancer cells, used to evaluate mitochondrial mass. *P < 0.05, and ****P < 0.0001.

Mitochondrial superoxide levels were further assessed using MitoSOX Red staining. The results showed that knockdown of either VEZT or SLC25A24 significantly increased MitoSOX Red fluorescence intensity (**Figures 14A–D**), indicating enhanced mitochondrial superoxide production and mtROS accumulation. In contrast, restoration of SLC25A24 expression in VEZT-knockdown cells markedly reduced MitoSOX Red fluorescence intensity (**Figures 14E, F**), suggesting that SLC25A24 partially alleviates the mitochondrial oxidative stress induced by VEZT depletion.

**Figure 15:**
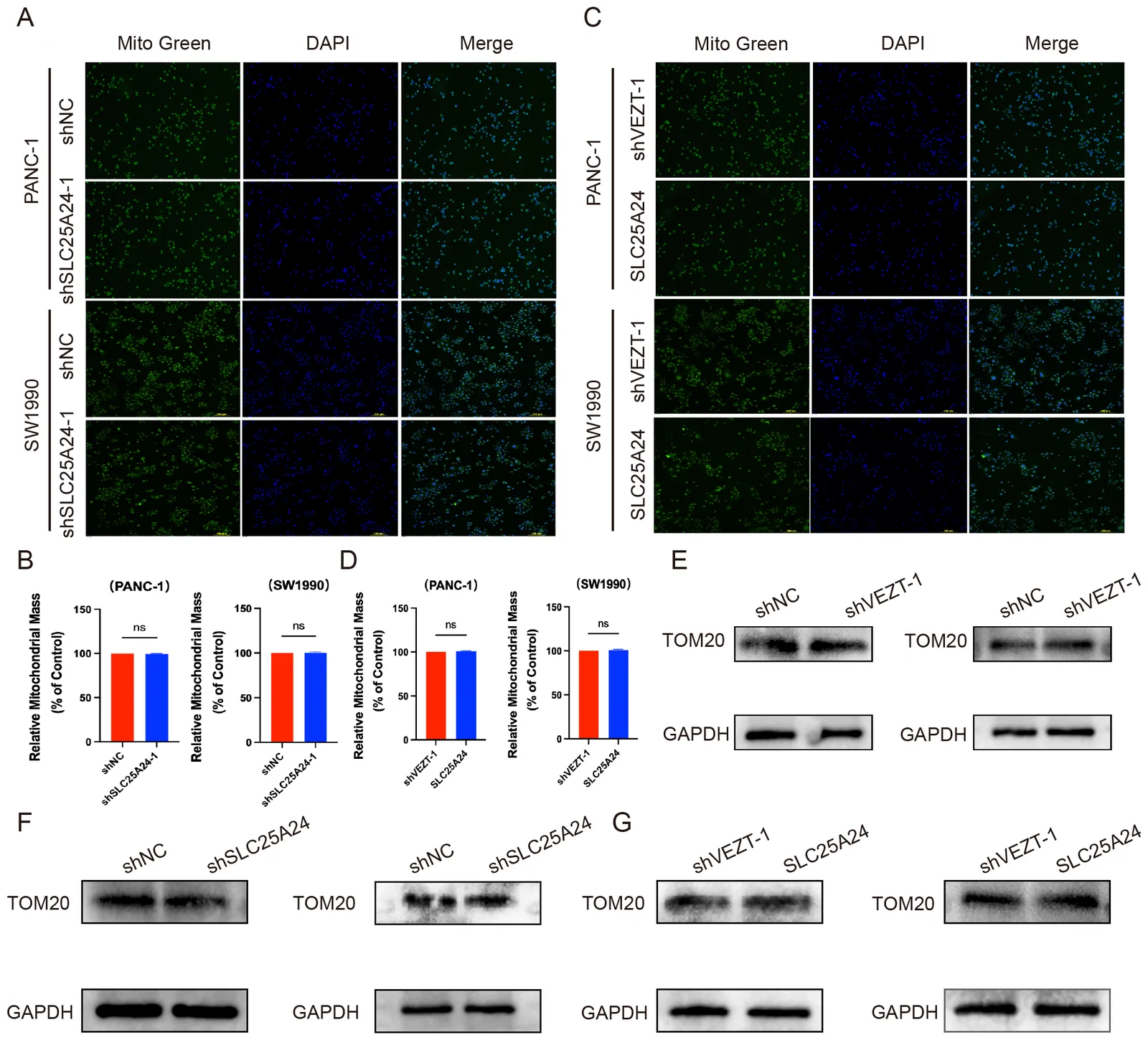
SLC25A24 knockdown and restoration do not significantly affect mitochondrial mass or content in pancreatic cancer cells. **(A, B)** Representative MitoTracker Green FM fluorescence images and quantitative analysis in control and SLC25A24-knockdown pancreatic cancer cells, used to assess mitochondrial mass. **(C, D)** Representative MitoTracker Green FM fluorescence images and quantitative analysis before and after restoration of SLC25A24 expression in VEZT-knockdown cells, used to assess mitochondrial mass. **(E)** Western blot analysis of the mitochondrial marker protein TOM20 in control and VEZT-knockdown PANC-1 and SW1990 pancreatic cancer cells. **(F)** Western blot analysis of TOM20 expression in control and SLC25A24-knockdown PANC-1 and SW1990 pancreatic cancer cells. **(G)** Western blot analysis of TOM20 expression before and after restoration of SLC25A24 expression in VEZT-knockdown PANC-1 and SW1990 pancreatic cancer cells.

Finally, mitochondrial mass and content were evaluated using MitoTracker Green FM staining and detection of the mitochondrial marker protein TOM20. The results showed that VEZT knockdown (**Figures 14G, H**), SLC25A24 knockdown, and restoration of SLC25A24 expression in VEZT-knockdown cells (**Figures 15A–D**) did not significantly alter MitoTracker Green FM fluorescence intensity or TOM20 protein expression levels (**Figures 15E–G**). These findings indicate that the VEZT/SLC25A24 axis primarily affects mitochondrial morphology, membrane potential, and oxidative stress, without significantly altering overall mitochondrial mass or content.

Taken together, VEZT may preserve mitochondrial network integrity and membrane potential while limiting excessive mtROS accumulation by maintaining SLC25A24 protein stability. Through this mechanism, VEZT sustains mitochondrial functional status, promotes pancreatic cancer cell survival, and suppresses apoptosis.

## 4 Discussion

Pancreatic cancer is a highly aggressive malignancy with an extremely poor prognosis, and the identification of novel therapeutic targets remains an urgent clinical need to improve patient outcomes (21). Although previous studies have suggested that VEZT may contribute to the malignant progression of gastric cancer, its biological functions and molecular mechanisms in pancreatic cancer have not been systematically elucidated (22). Pan-cancer analysis revealed that VEZT was aberrantly upregulated in multiple malignancies, including pancreatic cancer, and that its expression was significantly associated with patient prognosis across several tumor types. Consistent with these findings, VEZT was markedly overexpressed in pancreatic cancer tissues and cell lines and was closely associated with unfavorable clinical outcomes. Moreover, VEZT expression was positively correlated with tumor grade and clinical stage, suggesting that VEZT may contribute to pancreatic cancer progression and the development of aggressive tumor phenotypes.

Functional assays demonstrated that VEZT contributes to the maintenance of pancreatic cancer cell proliferation, migration, and survival, suggesting that it may participate in the establishment of malignant phenotypes in pancreatic cancer. To further investigate the underlying molecular basis, SLC25A24 was identified as a major candidate effector through correlation and prognostic analyses. SLC25A24 was highly expressed in pancreatic cancer and was associated with poor prognosis, higher tumor grade, and advanced pathological stage, indicating that it may contribute to pancreatic cancer progression.

The combined VEZT/SLC25A24 model exhibited favorable diagnostic and prognostic performance, suggesting that their coordinated overexpression may reflect a more aggressive tumor biological phenotype. However, the improvement in model performance over the corresponding single-gene models was limited. Moreover, the combined model was primarily developed using retrospective public datasets and has not yet been validated in independent clinical cohorts or prospective studies. Therefore, its clinical utility requires further evaluation in larger, multicenter cohorts and comparison with established clinical indicators, such as CA19-9, to determine whether it provides additional diagnostic or prognostic information. Notably, Cox regression analysis further showed that VEZT and SLC25A24 remained significantly associated with patient prognosis after adjustment for relevant clinicopathological factors, suggesting that both may have independent prognostic value in pancreatic cancer. These findings not only further support the close association of VEZT and SLC25A24 with malignant pancreatic cancer progression but also provide a basis for the potential clinical relevance of the VEZT/SLC25A24 axis.

Single-cell and spatial transcriptomic analyses further revealed that VEZT was predominantly enriched in epithelial and endothelial cells, whereas SLC25A24 was mainly expressed in epithelial cells. Their co-enrichment in epithelial cells provides cell type-level support for a potential functional association between these two molecules in pancreatic cancer cells. In addition, extensive ligand–receptor interactions were observed among epithelial cells, endothelial cells, T cells, and fibroblasts, suggesting that VEZT and SLC25A24 may be involved in the complex interplay among tumor cells, stromal cells, and immune cells.

With respect to immune infiltration, VEZT and SLC25A24 exhibited broadly similar correlation patterns. Both were positively correlated with the infiltration of resting memory CD4^+^ T cells but negatively correlated with CD8^+^ T cells and naïve B cells, suggesting that their elevated expression may be associated with alterations in immune cell composition and attenuation of antitumor immune responses in pancreatic cancer. However, these findings were mainly derived from transcriptome-based inference and correlation analyses and therefore do not establish that VEZT or SLC25A24 directly mediates specific cell–cell communication or immune regulatory processes. Their precise roles within the tumor microenvironment require further experimental validation.

Mechanistically, VEZT appeared to regulate SLC25A24 predominantly at the level of protein stability rather than transcription. Molecular docking, immunofluorescence colocalization, and co-immunoprecipitation collectively supported a potential protein association between VEZT and SLC25A24. Nevertheless, because SLC25A24 is mainly localized to the mitochondrial inner membrane, whereas VEZT is predominantly distributed in the nucleoplasm and cytoplasm, the current evidence is insufficient to demonstrate a direct physical interaction between the two proteins (23). They may become spatially proximal only within specific subcellular regions or during protein transport, or they may be indirectly associated through chaperones, scaffold proteins, or other intermediary molecules (24, 25). Cycloheximide chase assays further showed that VEZT knockdown accelerated SLC25A24 degradation and shortened its protein half-life, suggesting that VEZT may maintain SLC25A24 protein stability through either direct or indirect mechanisms.

SLC25A24, a member of the mitochondrial inner membrane carrier family, is involved in ATP/Mg^2+^ transport and ion homeostasis and plays essential roles in regulating mitochondrial membrane potential, energy metabolism, and cellular stress responses (8, 26, 27) As highly dynamic organelles, mitochondria are central regulators of cellular energy production, metabolic adaptation, and signal transduction. Accumulating evidence has demonstrated that mitochondria actively contribute to tumor initiation and progression through coordination of multiple biological processes (28) Metabolic reprogramming enables cancer cells to acquire sufficient energy and biosynthetic substrates through enhanced aerobic glycolysis, glutamine metabolism, and lipid metabolism, thereby supporting sustained proliferation, survival, and therapeutic resistance (29). Furthermore, genetic and epigenetic alterations in both mitochondrial and nuclear genomes can disrupt oxidative phosphorylation, tricarboxylic acid cycle dynamics, and cellular redox homeostasis, ultimately facilitating tumor development and progression (30). In addition, mitochondria serve as central hubs for intrinsic apoptotic signaling. Loss of mitochondrial membrane potential and increased mitochondrial membrane permeability trigger cytochrome c release and subsequent activation of the caspase cascade, whereas the BCL2/BAX axis functions as a critical regulator of this process (31, 32).

The present study further demonstrated that SLC25A24 promoted pancreatic cancer cell proliferation and migration while suppressing apoptosis. Notably, restoration of SLC25A24 expression in VEZT-knockdown cells partially reversed the impaired proliferation and migration and the enhanced apoptosis caused by VEZT depletion. Mitochondrial functional analyses further showed that knockdown of either VEZT or SLC25A24 resulted in mitochondrial loss of mitochondrial membrane potential, and accumulation of mitochondrial reactive oxygen species, accompanied by increased BAX expression and decreased BCL2 expression. Conversely, SLC25A24 overexpression in VEZT-knockdown cells partially restored mitochondrial membrane potential, reduced mitochondrial reactive oxygen species levels, and reversed the aberrant expression of BAX and BCL2. These findings further support an important role for SLC25A24 in VEZT-mediated maintenance of mitochondrial function and regulation of apoptosis.

Mitochondrial membrane potential is a major driving force for oxidative phosphorylation and ATP synthesis. Its reduction generally indicates impaired mitochondrial bioenergetic status, weakened proton-motive force, or abnormal membrane permeability and may further disrupt ATP production, calcium homeostasis, and mitochondrial quality-control processes (33). Meanwhile, excessive mtROS can oxidize mitochondrial DNA, membrane lipids, and respiratory-chain proteins, thereby further impairing electron transport efficiency and establishing a self-reinforcing cycle involving ROS accumulation, membrane potential loss, and mitochondrial dysfunction (34). Mitochondrial dynamics are closely linked to oxidative stress, and persistent mitochondrial damage is often accompanied by fragmentation of the mitochondrial network. Under severe conditions, such damage may trigger mitochondrial outer membrane permeabilization, cytochrome c release, and caspase-dependent apoptosis (35). Therefore, the concurrent loss of mitochondrial membrane potential, and elevation of mtROS indicates pronounced mitochondrial dysfunction and oxidative stress.

Notably, enrichment analyses further supported the close association of this molecular axis with mitochondrial function. GO and KEGG enrichment analyses of VEZT-associated genes revealed significant enrichment in glucuronate metabolism, xenobiotic metabolism, and ion transmembrane transport, as well as pathways involved in redox regulation and membrane potential maintenance. In contrast, SLC25A24-associated genes were primarily enriched in ion transport, membrane potential regulation, and calcium signaling-related processes. These findings suggest that the molecular network associated with the VEZT/SLC25A24 axis is closely linked to the regulation of cellular metabolic homeostasis and ion homeostasis, both of which are fundamentally connected to mitochondrial function and integrity (36).

In summary, the present study suggests that VEZT may preserve SLC25A24 protein stability, maintain mitochondrial membrane potential, and limit excessive mtROS accumulation, thereby alleviating mitochondrial dysfunction, reducing susceptibility to mitochondria-dependent apoptosis, and ultimately promoting pancreatic cancer cell survival and malignant progression.

Nevertheless, several limitations of this study should be acknowledged. First, the present findings were primarily based on *in vitro* cellular experiments and bioinformatics analyses and have not yet been validated *in vivo*. Therefore, the regulatory effects of the VEZT/SLC25A24 axis on pancreatic cancer growth, metastasis, and mitochondrial function, as well as the potential therapeutic value of targeting this axis, require further confirmation using subcutaneous xenograft, orthotopic transplantation, or other appropriate *in vivo* models. Second, the precise molecular mechanism by which VEZT regulates SLC25A24 protein stability remains incompletely understood. Given the differences in the subcellular localization of VEZT and SLC25A24, it remains unclear whether the two proteins interact directly, whether their association depends on intermediary proteins, and whether this process involves the ubiquitin–proteasome system or other post-translational modifications. These questions warrant further investigation. In addition, the relationship between the VEZT/SLC25A24 axis and the tumor immune microenvironment is currently supported mainly by correlation analyses and cell–cell communication predictions. Further validation using immunocompetent animal models, spatial multi-omics approaches, and cell type-specific intervention experiments is therefore required.

## 5 Conclusion

In summary, the present study demonstrated that both VEZT and SLC25A24 are significantly upregulated in pancreatic cancer and that their elevated expression is closely associated with unfavorable patient prognosis, suggesting their potential utility as prognostic biomarkers. Mechanistically, VEZT promotes pancreatic cancer progression by stabilizing SLC25A24 protein expression, thereby modulating mitochondrial function and suppressing apoptosis. Collectively, these findings suggest that the VEZT/SLC25A24 axis may contribute to the malignant progression of pancreatic cancer and may represent a potential target for therapeutic intervention.

## Data Availability

The raw data supporting the conclusions of this article will be made available by the authors, without undue reservation.

## References

[1] Stoop TF Javed AA Oba A Koerkamp BG Seufferlein T Wilmink JW . Pancreatic cancer. Lancet. (2025) 405:1182–202. doi: 10.1016/S0140-6736(25)00261-2 40187844

[2] Guo X Jing C Li L Zhang L Shi Y Wang J . Down-regulation of VEZT gene expression in human gastric cancer involves promoter methylation and miR-43c. Biochem Biophys Res Commun. (2011) 404:622–7. doi: 10.1016/j.bbrc.2010.12.026 21156161

[3] Spinner MA Pinter K Drerup CM Herman TG . A conserved role for Vezatin proteins in cargo-specific regulation of retrograde axonal transport. Genetics. (2020) 216:431–45. doi: 10.1534/genetics.120.303499 32788307 PMC7536845

[4] Hyenne V Louvet-Vallée S El-Amraoui A Petit C Maro B Simmler MC . Vezatin, a protein associated to adherens junctions, is required for mouse blastocyst morphogenesis. Dev Biol. (2005) 287:180–91. doi: 10.1016/j.ydbio.2005.09.004 16199027

[5] Ding H Liu C Sun Q Jiang Y Xu Q Zhu Z . Multitrait GWAS and functional validation reveal genetic loci for gastric cancer. Nat Commun. (2026) 17:4006. doi: 10.1038/s41467-026-70774-9 41833930 PMC13136404

[6] Li YS Chen YZ Guo XB Liu X Li LP . VEZT as a novel independent prognostic factor in gastric cancer. Cancer biomark. (2015) 15:375–80. doi: 10.3233/CBM-150476 25792470 PMC12965086

[7] Berridge MV Zobalova R Boukalova S Caicedo A Rushworth SA Neuzil J . Horizontal mitochondrial transfer in cancer biology: Potential clinical relevance. Cancer Cell. (2025) 43:803–7. doi: 10.1016/j.ccell.2025.03.002 40118050

[8] Harborne SP King MS Crichton PG Kunji ER . Calcium regulation of the human mitochondrial ATP-Mg/Pi carrier SLC25A24 uses a locking pin mechanism. Sci Rep. (2017) 7:45383. doi: 10.1038/srep45383 28350015 PMC5369052

[9] Osipov EM Strelkov SV . DDtrek: A pyMOL-based management system for 3D structural data series. ACS Omega. (2025) 10:21024–9. doi: 10.1021/acsomega.4c07417 40488029 PMC12138592

[10] Zhang Y Hua S Jiang Q Xie Z Wu L Wang X . Identification of feature genes of a novel neural network model for bladder cancer. Front Genet. (2022) 13:912171. doi: 10.3389/fgene.2022.912171 35719407 PMC9198295

[11] Wu L Cen C Ouyang D Zhang L Li X Wu H . Interpretable machine learning model for predicting early recurrence of pancreatic cancer: integrating intratumoral and peritumoral radiomics with body composition. Int J Surg. (2025) 111:8198–211. doi: 10.1097/JS9.0000000000003078 40717595 PMC12626599

[12] Zheng S Liu Y Troy Teo P Wu Y Zhang J Abazeed ME . A deep learning model for preoperative risk stratification of pancreatic ductal adenocarcinoma based on genomic predictors of liver metastasis. Eur J Cancer. (2025) 226:115608. doi: 10.1016/j.ejca.2025.115608 40628176

[13] Esengur OT Stevenson E Stecko H Lay NS Yang D Tetreault J . Assessing the impact of transition and peripheral zone PSA densities over whole-gland PSA density for prostate cancer detection on multiparametric MRI. Prostate. (2025) 85:612–24. doi: 10.1002/pros.24863 39996409

[14] Wang Z Liu Y Zhang X Wang C Tian J Zhao H . Construction of a nomogram for hypertriglyceridemic severe acute pancreatitis that includes metabolic indexes. Lipids Health Dis. (2025) 24:279. doi: 10.1186/s12944-025-02702-7 40926228 PMC12418621

[15] Ding Q Xu Q Hong Y Zhou H He X Niu C . Integrated analysis of single-cell RNA-seq, bulk RNA-seq, Mendelian randomization, and eQTL reveals T cell-related nomogram model and subtype classification in rheumatoid arthritis. Front Immunol. (2024) 15:1399856. doi: 10.3389/fimmu.2024.1399856 38962008 PMC11219584

[16] Kiviaho A Eerola SK Kallio HML Andersen MK Hoikka M Tiihonen AM . Single cell and spatial transcriptomics highlight the interaction of club-like cells with immunosuppressive myeloid cells in prostate cancer. Nat Commun. (2024) 15:9949. doi: 10.1038/s41467-024-54364-1 39550375 PMC11569175

[17] Wang X McManus M . Lentivirus production. J Vis Exp. (2009) (32):1499. doi: 10.3791/1499 19801965 PMC2865973

[18] Fishman JB Berg EA . Protein A and protein G purification of antibodies. Cold Spring Harb Protoc. (2019) 2019:10.1101/pdb.prot099143. doi: 10.1101/pdb.prot099143 30602558

[19] Miao Y Du Q Zhang HG Yuan Y Zuo Y Zheng H . Cycloheximide (CHX) chase assay to examine protein half-life. Bio Protoc. (2023) 13:e4690. doi: 10.21769/BioProtoc.4690 37323633 PMC10262206

[20] Wang H Cui C Li W Wu H Sha J Pan J . AGD1/USP10/METTL13 complexes enhance cancer stem cells proliferation and diminish the therapeutic effect of docetaxel via CD44 m6A modification in castration resistant prostate cancer. J Exp Clin Cancer Res. (2025) 44:12. doi: 10.1186/s13046-025-03272-3 39806412 PMC11730809

[21] Stickler S Rath B Hamilton G . Targeting KRAS in pancreatic cancer. Oncol Res. (2024) 32:799–805. doi: 10.32604/or.2024.045356 38686056 PMC11055996

[22] Xie D Shang L Peng L Li L . Up-regulation of VEZT by small activating RNA inhibits the proliferation, invasion and migration of gastric cancer cells. Biochem Biophys Res Commun. (2017) 482:542–8. doi: 10.1016/j.bbrc.2016.11.071 27856244

[23] Run C Yang Q Liu Z OuYang B Chou JJ . Molecular basis of MgATP selectivity of the mitochondrial SCaMC carrier. Structure. (2015) 23:1394–403. doi: 10.1016/j.str.2015.06.004 26165595 PMC4526376

[24] Pfanner N Warscheid B Wiedemann N . Mitochondrial proteins: from biogenesis to functional networks. Nat Rev Mol Cell Biol. (2019) 20:267–84. doi: 10.1038/s41580-018-0092-0 30626975 PMC6684368

[25] Araiso Y Tsutsumi A Qiu J Imai K Shiota T Song J . Structure of the mitochondrial import gate reveals distinct preprotein paths. Nature. (2019) 575:395–401. doi: 10.1038/s41586-019-1680-7 31600774

[26] Gao Y Peng Y Zhou Y Zhu J Fu S Chen Y . Mitochondrial gene SLC25A24 regulated anti-tumor immunity and inhibited the proliferation and metastasis of colorectal cancer by PKG1-dependent cGMP/PKG1 pathway. Int Immunopharmacol. (2025) 157:114664. doi: 10.1016/j.intimp.2025.114664 40334626

[27] Writzl K Maver A Kovačič L Martinez-Valero P Contreras L Satrustegui J . De novo mutations in SLC25A24 cause a disorder characterized by early aging, bone dysplasia, characteristic face, and early demise. Am J Hum Genet. (2017) 101:844–55. doi: 10.1016/j.ajhg.2017.09.017 29100094 PMC5673633

[28] Du H Xu T Yu S Wu S Zhang J . Mitochondrial metabolism and cancer therapeutic innovation. Signal Transduct Target Ther. (2025) 10:245. doi: 10.1038/s41392-025-02311-x 40754534 PMC12319113

[29] Vaupel P Schmidberger H Mayer A . The Warburg effect: essential part of metabolic reprogramming and central contributor to cancer progression. Int J Radiat Biol. (2019) 95:912–9. doi: 10.1080/09553002.2019.1589653 30822194

[30] Rossmann MP Dubois SM Agarwal S Zon LI . Mitochondrial function in development and disease. Dis Model Mech. (2021) 14:dmm048912. doi: 10.1242/dmm.048912 34114603 PMC8214736

[31] White MJ McArthur K Metcalf D Lane RM Cambier JC Herold MJ . Apoptotic caspases suppress mtDNA-induced STING-mediated type I IFN production. Cell. (2014) 159:1549–62. doi: 10.1016/j.cell.2014.11.036 25525874 PMC4520319

[32] Zhang A Wang Y Xue Q Yao J Chen L Feng S . Polystyrene microplastics disrupt kidney organoid development via oxidative stress and Bcl-2/Bax/caspase pathway. Chem Biol Interact. (2025) 419:111642. doi: 10.1016/j.cbi.2025.111642 40619079

[33] Osellame LD Blacker TS Duchen MR . Cellular and molecular mechanisms of mitochondrial function. Best Pract Res Clin Endocrinol Metab. (2012) 26:711–23. doi: 10.1016/j.beem.2012.05.003 23168274 PMC3513836

[34] Zorov DB Juhaszova M Sollott SJ . Mitochondrial reactive oxygen species (ROS) and ROS-induced ROS release. Physiol Rev. (2014) 94:909–50. doi: 10.1152/physrev.00026.2013 24987008 PMC4101632

[35] Bock FJ Tait SWG . Mitochondria as multifaceted regulators of cell death. Nat Rev Mol Cell Biol. (2020) 21:85–100. doi: 10.1038/s41580-019-0173-8 31636403

[36] Zorova LD Popkov VA Plotnikov EY Silachev DN Pevzner IB Jankauskas SS . Mitochondrial membrane potential. Anal Biochem. (2018) 552:50–9. doi: 10.1016/j.ab.2017.07.009 28711444 PMC5792320

